# Unraveling the photoredox chemistry of a molecular ruby

**DOI:** 10.1039/d5sc05170c

**Published:** 2025-09-10

**Authors:** Guangjun Yang, Georgina E. Shillito, Phillip Seeber, Oliver S. Wenger, Stephan Kupfer

**Affiliations:** a Institute of Physical Chemistry, Friedrich Schiller University Jena Lessingstraße 4 07743 Jena Germany; b University Computer Centre, Friedrich Schiller University Jena Am Johannesfriedhof 2 07743 Jena Germany; c Department of Chemistry, University of Basel St. Johanns-Ring 19 4056 Basel Switzerland stephan.kupfer@uni-jena.de

## Abstract

In contrast to well-studied 4d^6^ and 5d^6^ transition metal complexes such as the modern-day drosophila of photochemistry, Ru(ii)-tris(bipyridine), which often feature a typical triplet metal-to-ligand charge transfer emission in the nanosecond timescale, the photophysics of Cr(iii) complexes are drastically different. The 3d^3^ configuration of the chromium(iii) allows for an unusual spin-flip emission from the low-lying metal-centered (MC; ^2^T_1_ and ^2^E) states, exhibiting lifetimes up to the milliseconds to seconds timescale. In this fully computational contribution, the photophysical properties as well as the application of such long-lived excited states in the context of photoredox chemical transformations are investigated for the recently introduced [Cr(dqp)_2_]^3+^ [Cr(iii)-(2,6-bis(8′-quinolinyl)pyridine)_2_]^3+^, otherwise known as a type of molecular ruby. Our in-depth theoretical characterization of the complicated electronic structure of this 3d^3^ system relies on state-of-the-art multiconfigurational methods, *i.e.* the restricted active space self-consistent field (RASSCF) method followed by second-order perturbation theory (RASPT2). This way, the light-driven processes associated with the initial absorption from the quartet ground state, intersystem crossing to the doublet manifold as well as the spin-flip emission were elucidated. Furthermore, the applicability of the long-lived excited state in [Cr(dqp)_2_]^3+^ in photoredox chemistry, *i.e.* reductive quenching by *N*,*N*-dimethylaniline, was investigated by *ab initio* molecular dynamics (AIMD). Finally, the thermodynamics and kinetics of these underlying intermolecular electron transfer processes were analyzed in the context of semiclassical Marcus theory.

## Abstract 2

Photophysical and kinetic processes in a Cr(iii) spin-flip luminescent complex were elucidated using in-depth computational modelling. Use of the excited state in photoredox applications was also investigated using *ab initio* molecular dynamics.

## Graphical abstract

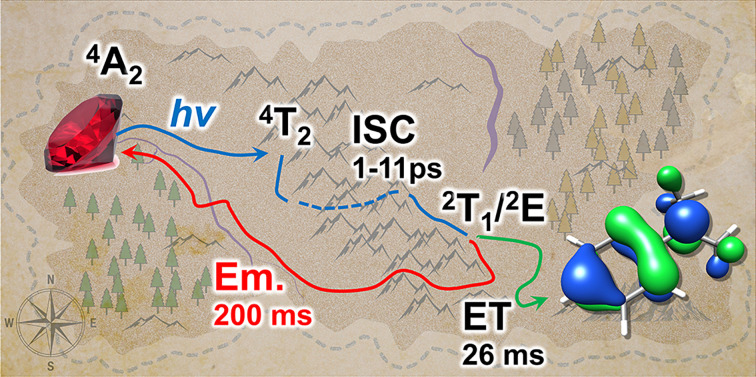

## 1 Introduction

The conversion of solar energy directly into electricity and chemical fuels, such as hydrogen or hydrocarbons, stands as a primary goal for humankind in order to transform our energy sector towards sustainability. In recent decades, luminescent transition metal complexes have been thoroughly investigated and widely utilized across diverse fields.^1–8^ Traditionally, there has been significant dependence on compounds containing 4d and 5d transition metals such as Ru(ii), Ir(iii), Os(ii) or Pt(II).^9–23^ The combination of favorable redox properties, efficient light-harvesting capabilities and stability to light, heat, and pH make them desirable candidates for light-harvesting or (photo)catalytic processes. Furthermore, on account of the large t_2g_–e_g_ ligand field splitting in octahedral 4d and 5d polypyridyl complexes and heavy-atom facilitated spin–orbit coupling (SOC), the lowest energy excited state is often emissive, long-lived and of triplet metal-to-ligand charge transfer (^3^MLCT) character. Metal centered (MC) states, whose population typically results in rapid non-radiative deactivation to the ground state and potentially even photodegradation, are high in energy and usually not easily accessible. In contrast the excited states of polypyridyl complexes of 3d metals such as Fe(ii) and Co(iii) are normally short-lived and non-emissive,^24–28^ due to low-lying and rapidly accessible MC states. Electronically, this drastically altered situation stems from the primogenic effect, *i.e.*, the smaller overlap of the 3d-orbitals with the ligand's lone-pair orbitals.^29^

**Fig. 1:**
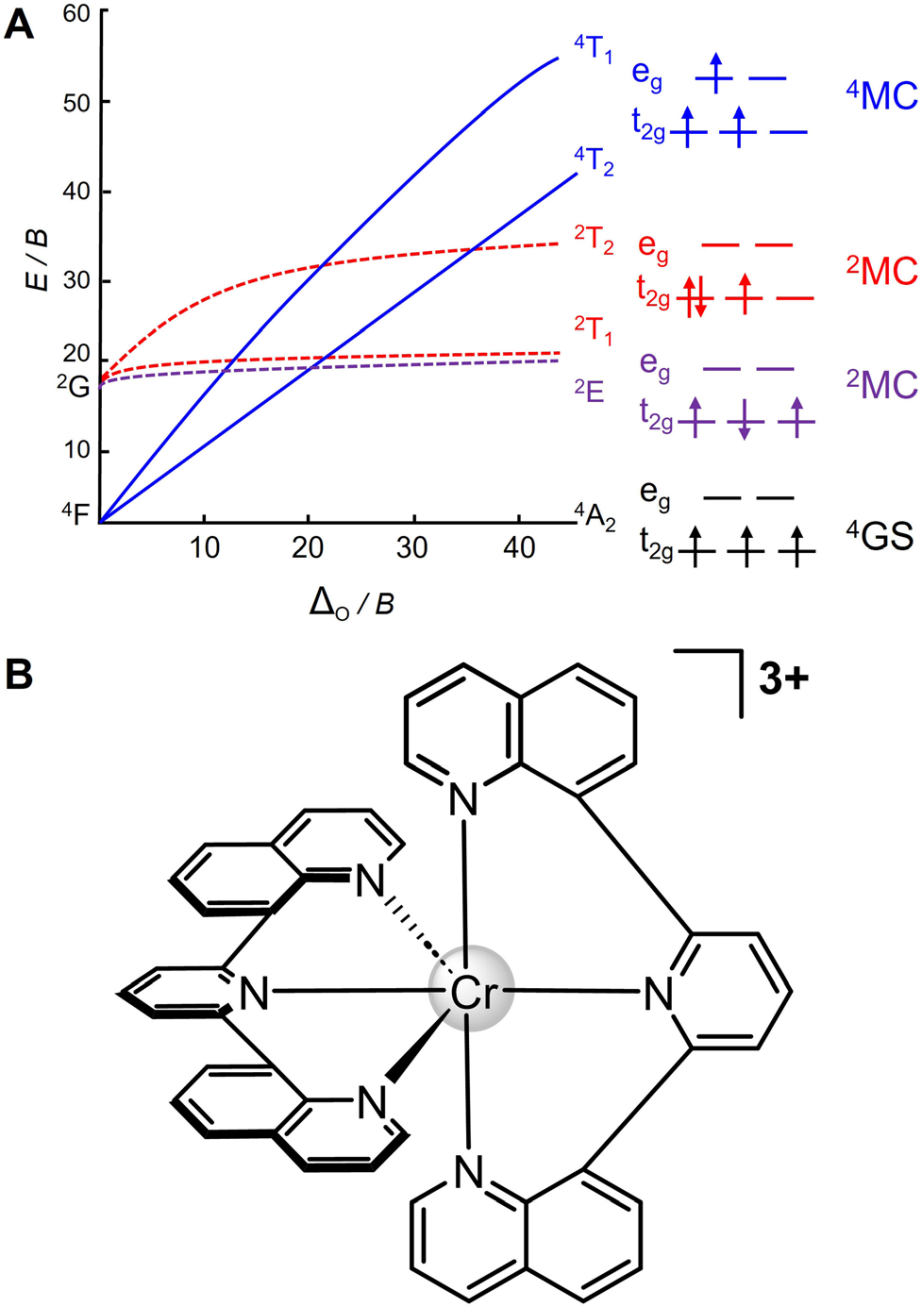
(A) Tanabe–Sugano diagram of d^3^ octahedral ligand field and, (B) structure of [Cr(dqp)_2_]^3+^.

In contrast certain, carefully designed Cr(iii) complexes – with a quartet 3d^3^ ground state configuration – are often strongly emissive and can exhibit unusually long excited state lifetimes. This behavior is attributed to a property known as spin-flip emission.^5,30–32^ In the case of this curious phenomenon, the excited state and the ground state share the same electronic configuration, with the only difference being the spin of one electron, see Fig. 1A.

In the case of the present Cr(iii)-based family of complexes (see [Cr(dqp)_2_]^3+^ as shown in Fig. 1B), this unique electronic structure leads to phosphorescence from a ^2^MC state back to the ^4^MC ground state. These emissive states are in stark contrast to the common emissive ^3^MLCT states, *i.e.* in second- and third-row transition metal coordination compounds with *e.g.*, d^6^ and d^8^ configurations. Notably, traditional ligands such as 1,2-ethylenediamine (en) or 2,2′;6′,2′′-terpyridine (tpy) are not suited to rationalize spin-flip luminescence in 3d^3^ systems, such as Cr(iii),^33,34^ due to their substantial divergence from a perfectly octahedral geometry (by approximately 7° to 11°). In consequence, a relatively weak ligand field is formed based on the inefficient overlap of the respective orbitals forming the coordination environment. Thus, the proximity of the ^4^MC excited state (^4^T_2_) and ^2^MC states (^2^E and ^2^T_1_), as shown in Fig. 1A, promotes back-intersystem crossing (bISC) from the ^2^E/^2^T_1_ states to the Jahn–Teller distorted ^4^T_2_ states. This process is known to reduce the excited state lifetime by population of undesirable pathways associated with non-radiative decay or even (photo)degradation of the coordination environment.^30,35^ Therefore, the pursuit of enhancing ligand field splitting by judicious ligand selection to construct a perfectly octahedral structure has become a focal point of interest for synthetic and theoretical chemists in the past ten years.^31,34,36–45^ For instance, the Heinze group recently reported the coordination of two six-membered 2,6-bis(2-pyridylmethyl)pyridine (bpmp) and *N*,*N*′-dimethyl-*N*,*N*′-dipyridine-2-ylpyridine-2,6-diamine (ddpd) chelates ([Cr(bpmp)_2_]^3+^ and [Cr(ddpd)_2_]^3+^). The careful ligand selection not only provides less distorted excited states, thereby limiting the non-radiative decay but also strong ligand field splitting to prevent undesirable ^4^T_2_–^2^E/^2^T_1_ bISC. Both Cr(iii) complexes display significantly long lifetimes (bpmp: 1360 μs; ddpd: 889 μs) and high luminescence quantum yields (bpmp: 12.3%; ddpd: 11.0%) in deaerated aqueous solutions at room temperature.^39,45^ Notably, the nuclear charge of the central metal (ion) is key to balancing the energy levels of the involved locally excited MC states and charge transfer states. As suggested by recent studies on several 3d^3^ complexes, Cr(iii) seems to represent a unique sweet spot in the periodic table of elements which facilitates population of rather low-lying ^4/2^MC states involved in the spin-flip-based lifetime elongation. In contrast, introduction of V(ii) and Mn(iv) centers – with a comparable 3d^3^ configuration –alters the complex excited state landscape in favor of MLCT and LMCT (ligand-to-metal charge transfer) processes, respectively.^32,46–48^

In the present contribution, we carefully evaluate the photophysical and photoredox properties associated with spin-flip luminescence of a Cr(iii) complex [Cr(iii)-(2,6-bis(8′-quinolinyl)pyridine)_2_]^3+^ ([Cr(dqp)_2_]^3+^), see Fig. 1B, which was first reported by Piguet's team in 2019.^41^ Since then, this Cr(iii) spin-flip system, which features an excited state lifetime of 1.2 ms (in water) at room temperature, was investigated in the context of developing material capable of circularly polarized luminescence in the near-infrared (NIR) region and polarized photonic devices based on earth-abundant metals.^41^ Very recently, the photoredox properties of this long-lived spin-flip species in combination with a *N*,*N*-dimethylaniline (DMA) electron donor were investigated in the Wenger group.^36,49^ Based on these previous works, the focus of the present contribution is set on unraveling the unique photophysical properties of Cr(iii) spin-flip complexes and their application in light-driven redox chemistry by means of theoretical modelling with [Cr(dqp)_2_]^3+^ as a representative complex for the molecular ruby family of compounds.

In order to provide an unbiased description of the involved electronic states, such as the ^4^MC and ^2^MC states of interest (as well as MLCT and LMCT states), multiconfigurational methods are necessary, which allow for the consideration of static electron correlation, particularly associated with the d-orbitals. Here, we utilize the state-of-the-art restricted active space self-consistent field (RASSCF) method followed by second-order perturbation theory (RASPT2). This methodology (RASPT2//RASSCF) allows for an elaborate description of both static and dynamic electron correlation for medium-sized coordination compounds.^50–52^ In particular, splitting of the active space into three subspaces enables the construction of sizable active spaces which further accounts for addressing the double d-shell effect – related to the partial contribution of energetically close-lying 4d orbitals. In addition to modelling of the light-driven processes, we also estimate the kinetics of intersystem crossing (ISC) as well as emissive processes among the quartet and doublet states of interest. Furthermore, these demanding multiconfigurational calculations are compared to cost-efficient density functional and time-dependent density functional theory simulations (DFT and TDDFT). Surprisingly, the complex electronic structure of [Cr(dqp)_2_]^3+^ within the quartet ground state as well as involving weakly dipole-allowed MC and strongly dipole-allowed LMCT transitions are sufficiently described by cost-efficient TDDFT simulations as benchmarked with respect to RASPT2 and experimental reference data. Finally, the photoredox processes between the excited Cr(iii) chromophore and a DMA electron donor are unraveled at the molecular level, *i.e.* associated with the thermodynamics and the kinetics of the intermolecular electron transfer reaction. To this aim, we rely on *ab initio* molecular dynamics (AIMD) to describe the light-driven electron transfer kinetics in the frame of semi-classical Marcus theory. Based on our AIMD simulations and subsequent Marcus analysis, a comparably fast intermolecular electron transfer from the DMA donor to the excited [Cr(dqp)_2_]^3+^ was determined, while the reduction of the molecular ruby involves partial reduction of the chromium as well as of the ligand sphere.

## 2 Computational details

The DFT and TDDFT calculations addressing structural and electronic properties in both the ground and excited state of [Cr(dqp)_2_]^3+^ were performed utilizing the Gaussian 16 program^53^ with the B3LYP^54–59^ exchange correlation (XC) functional. All multiconfigurational (RASPT2//RASSCF) calculations were performed in MOLCAS 8.4 (ref. 60) using the geometry (*D*_2_ point group) as obtained at the DFT level of theory. AIMD simulations were performed using the CP2K 2024.1 software^61,62^ to assess the intermolecular ET processes in solution (acetonitrile, DMA) in a fully periodic fashion using the BLYP functional.^55,57,63^ A detailed description of the applied computational protocols is provided in the SI.

## 3 Results and discussion

### 3.1 Initial photoactivation

**Fig. 2:**
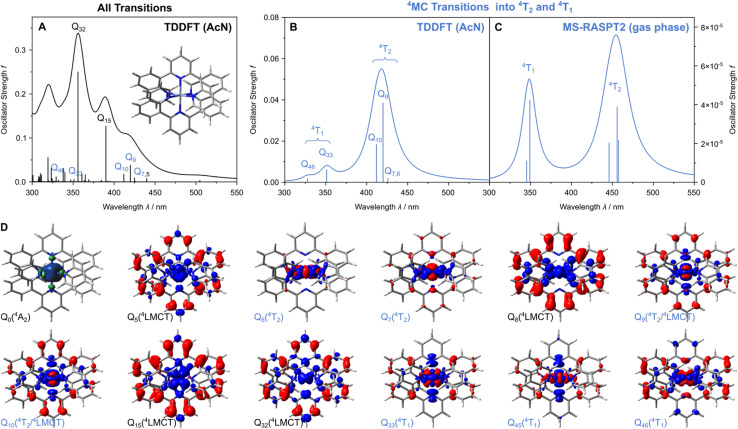
(A) Simulated UV-vis absorption spectrum of [Cr(dqp)_2_]^3+^ as predicted at the TDDFT level of theory in acetonitrile (AcN). Simulated transitions are broadened by Lorentzian functions with a full width at half maximum of 0.1 eV; state labels according to color scheme of Fig. 1. (B) and (C) Contribution of quartet metal-centered (^4^MC) transitions, *i.e.*, associated with low-energy ^4^T_2_ and high-energy ^4^T_1_ states at the TDDFT and MS-RASPT2 levels of theory, respectively. (D) Spin density of the quartet ground state (^4^A_2_) as well as charge density difference plots for selected ^4^LMCT and ^4^MC (^4^T_2_ and ^4^T_1_) transitions; charge transfer occurs from red to blue.

The DFT optimization of [Cr(dqp)_2_]^3+^ yields a *D*_2_ structure with a quartet ground state (^4^A_2_, Fig. 1A). The spin density which allows the unpaired electron density to be visualized (Fig. 2D) reveals a (t_2g_)^3^ electronic configuration, *i.e.* a quartet metal-centered (^4^MC) ground state where the 3d_*xy*_, 3d_*xz*_ and 3d_*yz*_ orbitals are each singly occupied.^36,45,64^ Notably, we apply in the following the typically utilized nomenclature to label the respective electronic states involved in the photophysics of pseudo-octahedral Cr(iii) complexes, which stems from the *O*_h_ point group. The previously reported UV-vis absorption spectrum of [Cr(dqp)_2_]^3+^ in acetonitrile (AcN) features the typical absorptions at 335 and 377 nm as well as a weak shoulder tailing to 450 nm.^36^ Analogously to the experiment, the electronic absorption spectrum of [Cr(dqp)_2_]^3+^, as obtained at the TDDFT level of theory and in AcN (polarizable continuum model), is dominated by two strongly dipole-allowed quartet ligand-to-metal charge transfer (^4^LMCT) excitations (Q_15_ and Q_32_), see Fig. 2A. In order to assign the nature of the respective electronic transitions we utilize charge density difference plots, this approach provides a straightforward tool to visualize changes of electron density upon excitation, *i.e.* electron hole and excited electron, in a single picture, see Fig. 2D. These excitations involve a population transfer mainly from the high-lying π_dqp_ orbitals of both dqp ligands into the t_2g_ orbitals (3d_*xy*_, 3d_*xz*_ and 3d_*yz*_). The predicted excitation energies of 3.18 and 3.48 eV (390 and 356 nm) are in good agreement with the experimental results (see Fig. 2A and Table S3). In addition, weakly-dipole allowed transitions associated with the ^4^T_1_ states (excited ^4^MC species) are predicted at 351 and 328 nm (Q_33_ and Q_46_). Finally, TDDFT predicts two pure ^4^T_2_ (^4^MC) states (into Q_6_ and Q_7_) as well as two mixed states stemming from a linear combination of the third ^4^T_2_ state and one ^4^LMCT state (into Q_9_ and Q_10_). The excitation wavelengths of these ^4^A_2_ → ^4^T_2_-transtions, ranging from 435 to 412 nm (Fig. 2 and Table S3), are in good agreement with the weak shoulder near 450 nm observed in the experimental UV-vis absorption spectrum.^36^ Notably, this superposition of ^4^T_2_ and ^4^LMCT character was also reported by the Heinze group for [Cr(bpmp)_2_]^3+^ and [Cr(ddpd)_2_]^3+^.^39,65^

**Fig. 3:**
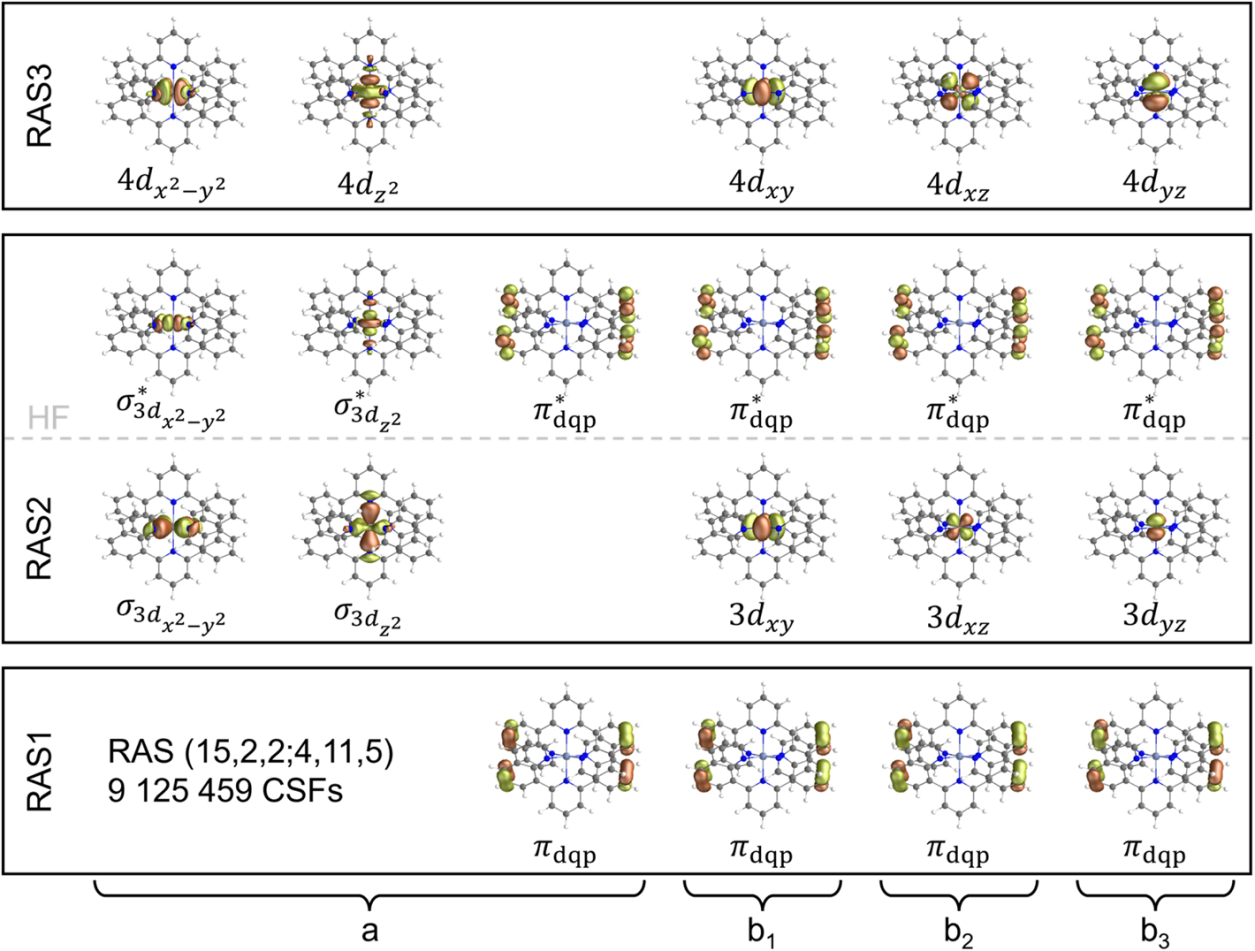
Molecular orbitals for the RAS (15,2,2;4,11,5) used in the state average procedure covering the lowest eight quartet and the lowest eight doublet roots of [Cr(dqp)_2_]^3+^; two in each irreducible representation. The partitioning with respect to the RAS1, RAS2 and RAS3 subspaces is indicated as well as the orbital occupation within the Hartree–Fock (HF) reference wavefunction with each t_2g_ orbital being singly occupied within the quartet ground state. The active space spans over 9 125 459 configuration state functions (CSFs).

In the following, we benchmark these TDDFT simulations against our multi-state RASPT2 results as obtained by a restricted active space (15,2,2;4,11,5). This active space spans over more than 9 million configuration state functions (CFSs) and comprises of two pairs of *σ*/σ* orbitals stemming from the linear combinations of the e_g_ orbitals (3d_*x*^2^−*y*^2^_ and 3d_*z*^2^_) and the respective lone-pairs of the two dqp ligands, the three t_2g_ orbitals (3d_*xy*_, 3d_*xz*_ and 3d_*yz*_), four pairs of 
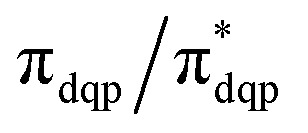
 orbitals as well as the five 4d orbitals to account for the double d-shell effect, see Fig. 3. RASPT2 simulations were performed in quartet and doublet multiplicity to address the relevant ^4^LMCT, ^4^ILCT, ^4^MC states and ^2^MC states (Tables S4 and S5); see SI for details regarding the computational setup.

Consistent with the DFT picture, the electronic ground state exhibits a ^4^A_2_ character (see Table S4), which features surprisingly a rather small multiconfigurational character as given by the reference weight of 86% for the Hartree–Fock determinate. Furthermore, three low-lying ^4^MC (^4^T_2_) states are predicted at 2.71, 2.72 and 2.78 eV (457, 456 and 446 nm), respectively. The weakly dipole-allowed excitations into these ^4^MC states are in excellent agreement with the experimentally observed shoulder at approximately 450 nm. The higher energy ^4^T_1_ (^4^MC) state also splits into three energy levels based on the pseudo-octahedral *D*_2_ symmetry. Energetically, the weakly dipole-allowed excitations are almost degenerate, at 349 and 345 nm (3.55 and 3.59 eV), while another excitation at slightly higher energy is dipole-forbidden (at 3.83 eV, 324 nm), see Fig. 2C and Table S4. Finally, the lowest energy ^4^ILCT state is obtained at 299 nm (4.14 eV); notably, this state features significant contributions of double excitations (DE), which are not covered by the TDDFT scheme, see Fig. S2c and Table S4.

Comparison of the multiconfigurational results for the low-lying ^4^T_2_ as well as for the higher lying ^4^T_1_ states with the TDDFT results reveals that, surprisingly, the B3LYP functional provides a balanced description of the electronic transitions associated with the ^4^MC states of interest. In case of the three ^4^T_2_ states, MS-RASPT2 predicts excitation energies of 2.71, 2.72 and 2.78 eV, while the TD-B3LYP energies are slightly overestimated by 0.04–0.1 eV (gas phase: 2.81, 2.88 and 2.92 eV, Table S2). In a similar fashion, the TDDFT and RASPT2 predicted ^4^T_1_ energies are in very good agreement (3.49, 3.74 and 3.75 eV *vs.* 3.55, 3.59 and 3.83 eV, Table S2). Noteworthily, the energy levels of these metal-centered states are (almost) insensitive to the solvent environment (AcN, Table S3). Therefore, and in summary, both TDDFT and MS-RASPT2 provide a balanced description of the energetics of the (quartet) ligand field states in [Cr(dqp)_2_]^3+^.

### 3.2 Intersystem crossing channels and spin-flip emission

**Fig. 4:**
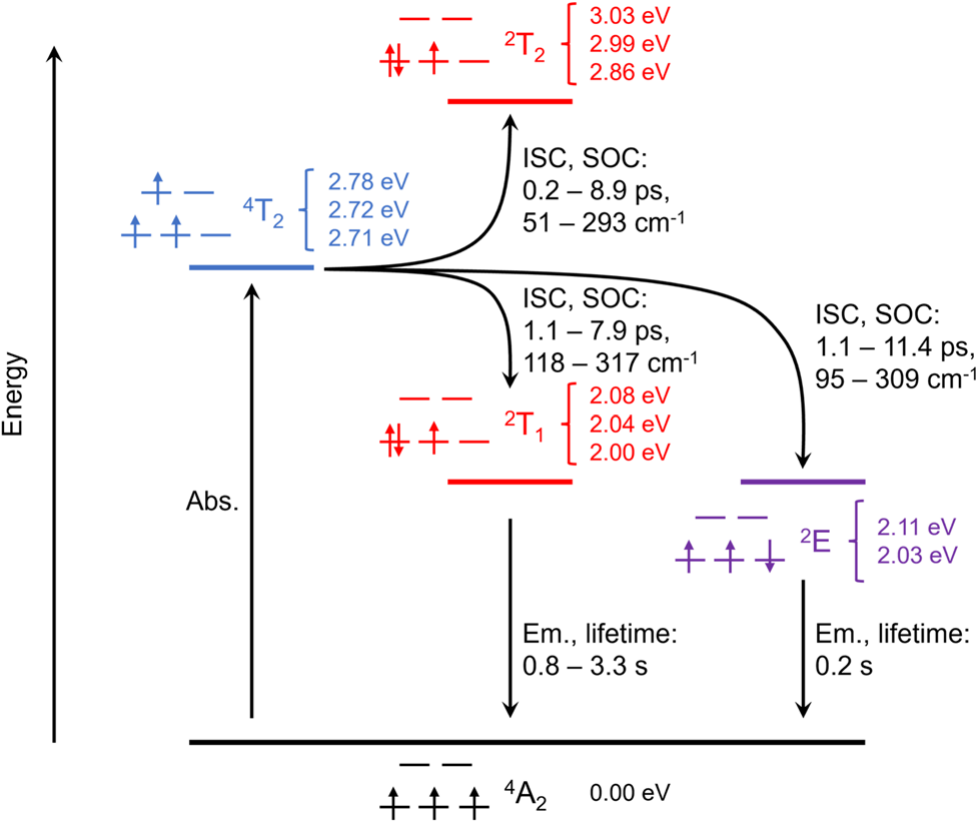
Jablonski scheme visualizing excited state processes in [Cr(dqp)_2_]^3+^ associated with intersystem crossing (ISC) from the quartet to the doublet manifold and radiative phosphorescence lifetimes as obtained based on the multiconfigurational results.

In the following section, we evaluate the kinetics of the ISC channels from the ^4^MC excited states (^4^T_2_) to the energetically close lying ^2^MC states (*i.e.*^2^T_2_, ^2^T_1_ and ^2^E) and the relaxation processes associated with the spin luminescence. Typically ISC in transition metal complexes occurs within few picoseconds or even on the sub-picosecond timescale based on pronounced relativistic effects introduced by the central metal atom (or other heavy atoms in the ligands sphere).^66–68^ The present Cr(iii) complex features a quartet ground state (^4^A_2_) and the initially populated excited states upon irradiation in the visible region are of ^4^T_2_ character, as discussed in Section 3.1. In addition to these quartet states, MS-RASPT2 was also utilized to investigate the low-lying doublet states of [Cr(dqp)_2_]^3+^. Notably, the equilibrium structures of the quartet ground state and of the emissive spin-flip doublet state are almost identical.^36^ Thus, the doublet energy levels, spin–orbit couplings (SOCs) and relaxation kinetics are obtained exclusively within the Franck–Condon region (^4^A_2_ ground state structure). The RASPT2 picture, predicted for the doublet (^2^MC) states, is consistent with the Tanabe–Sugano diagram, see Fig. 1A and Table S5. Two ^2^E states are predicted at 2.03 and 2.11 eV (611 and 588 nm), which are energetically close to the three predicted ^2^T_1_ states at 2.00, 2.04 and 2.08 eV (620, 608 and 596 nm), respectively. Furthermore, the three ^2^T_2_ states are found slightly higher in energy, *i.e.*, at 2.86, 2.99 and 3.03 eV (434, 415, 409 nm), respectively, see Fig. 4.

To treat both relativistic effects and electron correlation, the exact spin-free (SF) components and spin-dependent first-order Douglas–Kroll–Hess Hamiltonian^69^ are combined to calculate the spin–orbit couplings (SOCs) among the states of interest at the MS-RASPT2 level of theory. This way, spin–orbit (SO) states are constructed as linear combinations based on the individual SF states – the respective electronic states of quartet and doublet multiplicity. Thereby, the mixing between the otherwise orthogonal SF quartet and doublet states is reflected by their SOC, *i.e.* 〈*Q*_*j*_|*Ĥ*_SOC_|*D*_*i*_〉. The electronic absorption spectrum related to the ^4/2^MC states of interest within the SO picture (Fig. S3) is almost identical to the SF representation as shown in Fig. 2C. In order to evaluate the rate of ISC, the energies as well as the SOCs among the involved quartet and doublet states are essential; herein, we focus on the SOCs between the low-lying ^4^MC and ^2^MC states associated with the spin-flip of interest. Since the ^4^T_1_ states are less stable than the ^4^T_2_ states and undergo rapid internal conversion (IC) to the ^4^T_2_ states and subsequent vibrational cooling within 1–10 ps,^70^ ISC from the high-energy ^4^T_1_ states (3.55–3.83 eV, Table S4) is not considered in the present study.

In the case of [Cr(dqp)_2_]^3+^ our computational results reveal that the ^4^T_2_ states are strongly coupled with the low-lying ^2^T_1_ and ^2^E states (95–317 cm^−1^) as well as the higher-lying ^2^T_2_ states (51–293 cm^−1^). In comparison to other 3d transition metal complexes, the magnitude of these SOCs is rather large, which reflects the metal-centered character of the quartet donor and doublet acceptor states. Based on the energies and the SOCs among the ^4/2^MC states, ISC rates were obtained for both energetically uphill (^4^T_2_ → ^2^T_2_) and downhill (^4^T_2_ → ^2^T_1_ and ^4^T_2_ → ^2^E) processes, see eqn (1) and (2) in the SI. As shown in Fig. 4, in all cases considered herein, ultrafast ISC proceeds with rate constants in the range of 10^11^ to 10^12^ s^−1^ (0.2 to 11.4 ps), see Table 1 for details. These computational results clearly show that all ^2^MC states (^2^T_2_, ^2^T_1_ and ^2^E) are rapidly accessible upon ISC from the ^4^T_2_ states. Subsequently, internal conversion proceeds which leads presumably to an equal population of the quasi isoenergetic ^2^T_1_ and ^2^E states.

**Table 1:** Calculated ISC rate constants (top, in s^−1^ × 10^11^) as well as spin–orbit coupling elements 〈*Q*_*j*_|*Ĥ*_SOC_|*D*_*i*_〉 (bottom and in parenthesis, in cm^−1^), between prominent excited quartet and doublet states of [Cr(dqp)_2_]^3+^. All results were obtained at the RASPT2 level of theory

SOC *k*_ISC_	^2^T_1_ (2.00 eV)	^2^E (2.03 eV)	^2^T_1_ (2.04 eV)	^2^T_1_ (2.08 eV)	^2^E (2.11 eV)	^2^T_2_ (2.86 eV)	^2^T_2_ (2.99 eV)	^2^T_2_ (3.03 eV)
^4^T_2_ (2.71 eV)	0 (0)	9.46 (309)	2.51 (158)	1.60 (118)	2.81 (149)	18.12 (113)	12.61 (154)	0 (0)
^4^T_2_ (2.72 eV)	9.01 (317)	0.87 (95)	1.56 (126)	0 (0)	5.76 (216)	32.60 (146)	0 (0)	1.13 (51)
^4^T_2_ (2.78 eV)	1.27 (128)	1.58 (138)	0 (0)	3.58 (194)	5.55 (231)	0 (0)	16.30 (140)	53.30 (293)

The constructed Jablonski diagram (Fig. 4) indicates that the coordination of the dqp ligands to the metal is associated with an almost ideal overlap between e_g_ orbitals and the lone-pairs of the ligands, which is reflected by the almost perfect octahedral geometry (see bite angles in Table S1). This way, a strong ligand field splitting, which raises the ^4^T_2_ states far above the ^2^E and ^2^T_1_ states is established. Thermodynamically, the population of ^2^T_1_ and ^2^E states is most favorable with driving forces of approximately −0.7 eV. This way, bISC to the quartet states (^4^T_2_) is hampered, which leads to an enhanced phosphorescence quantum yield.

**Fig. 5:**
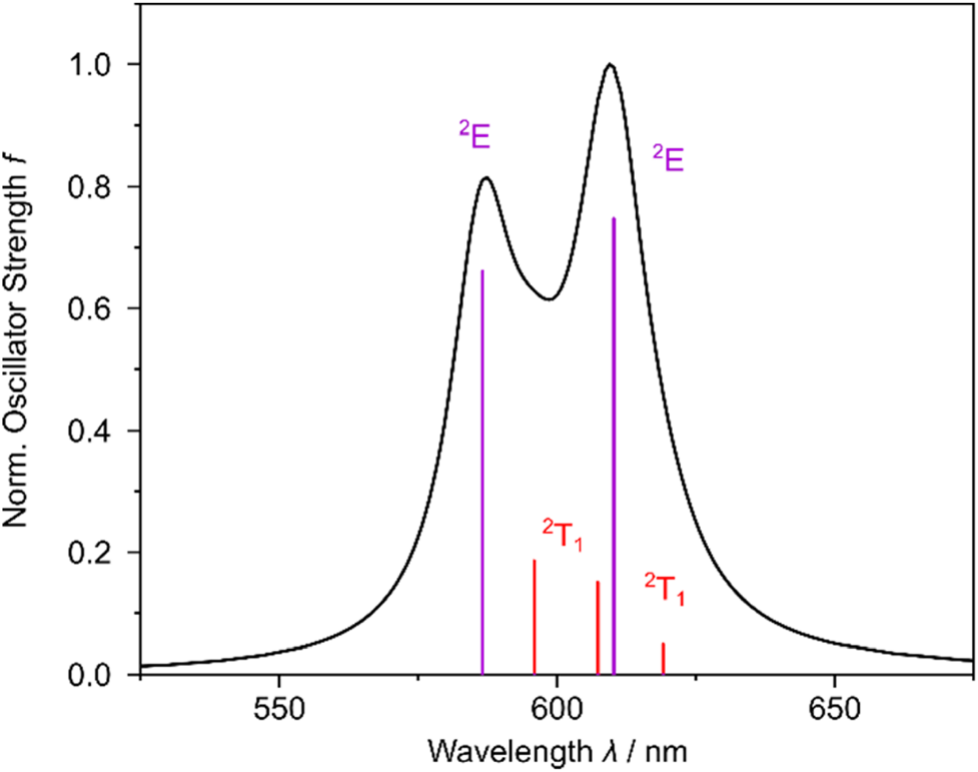
Simulated emission spectrum of [Cr(dqp)_2_]^3+^ based on the MS-RASPT2 data.

Finally, the population of the emissive ^2^E/^2^T_1_ states enables radiative decay (*k*_P_) to the (quartet, ^4^A_2_) ground state – or more precisely emission based on the energies and transition dipole moments between the respective SO states. As ISC to the ^2^T_1_ (1.1–7.9 ps) and ^2^E states (1.1–11.4 ps) occurs at almost identical timescales, a Boltzmann distribution of the respective microstates is expected. In consequence, the simulated emission spectrum is comprised of contributions from all three ^2^T_1_ and both ^2^E states. However, the Boltzmann population of the respective ^2^E and ^2^T_1_ states was approximated to be equal among the five quasi-isoenergetic microstates. This simplification was employed as no state-specific relaxation was performed, which would be essential to obtain reliable Boltzmann factors for each state, respectively. As shown in Fig. 5, two close-lying emissions bands at 609 and 588 nm (see Fig. 5), which are mainly associated with “true” spin-flip emission (^2^E → ^4^A_g_) emission at 611 and 588 nm (at 2.03 and 2.11 eV).

Emission from the ^2^T_1_ states (at 596, 609 and 620 nm, 2.00, 2.04, 2.08 eV) is less prominent due to their small transition dipole moments (see eqn (3) in the SI). This finding is also reflected by the calculated radiative lifetimes of 0.2 s in the case of the ^2^E states and even and 0.8–3.2 s for the ^2^T_1_ states, see Table 2. These computational results are in reasonable agreement with the experimentally observed emission of [Cr(dqp)_2_]^3+^, which features two narrow emission bands at 747 and 724 nm (1.66 and 1.71 eV), which were assigned to the lowest energy ^2^E and ^2^T_1_ states, respectively.^36,41^ We attribute the overestimation of the simulated emission energies (roughly +0.35 eV) mainly to the lack of excited state structural relaxation within our computational approach. In slight contrast to the previous assignment, our scalar relativistic MS-RASPT2 simulations suggest that both emission bands are related to ^2^E states, *i.e.*, due to their larger transition dipole moments (to the quartet ground state) as well as based on their shorter excited state lifetimes (see Fig. 4 and Table 2).

**Table 2:** Simulated radiative phosphorescence rates *k*_P_ (s^−1^) between low-lying doublet excited states (^2^E and ^2^T_1_) and the quartet ground state of [Cr(dqp)_2_]^3+^ and associated radiative lifetimes (*τ*_Rad_ = 1/*k*_P_ in s)

	^2^T_1_ (2.00 eV)	^2^E (2.03 eV)	^2^T_1_ (2.04 eV)	^2^T_1_ (2.08 eV)	^2^E (2.11 eV)
*k*_P_	0.31	4.80	1.25	0.98	4.59
1/*k*_P_	3.28	0.21	0.80	1.02	0.22

### 3.3 Intermolecular electron transfer

Based on our in-depth quantum chemical simulations, which allowed us to unravel the light-driven intramolecular processes and the spin-flip emission of the present molecular ruby system, we aim to extend our understanding in the context of its photoredox chemistry, *i.e.*, in the presence of a reductive quencher. Previously, Wenger *et al.* provided valuable insights into the driving force dependence of photoinduced ET between [Cr(dqp)_2_]^3+^ and a series of electron donors.^36,71^ The quenching rate was investigated through Stern–Volmer kinetics, using time-resolved luminescence and UV-vis transient absorption spectroscopy. Building on these recent studies, our quantum chemical and *ab initio* molecular dynamics (AIMD) simulations enable us to derive detailed structure–property relationships with respect to the underlying intermolecular electron transfer processes between the spin-flip excited Cr(iii) complex and the electron donor in solution (AcN). Here, we focus our computational efforts exclusively on the reductive quenching by *N*,*N*-dimethylaniline (DMA) as a representative organic electron donor. The derived theoretical understanding of the thermodynamic and kinetic properties is crucial to harvest the energy of millisecond-lived excited states in Cr(iii) spin-flip complexes in future photocatalytic applications.

**Fig. 6:**
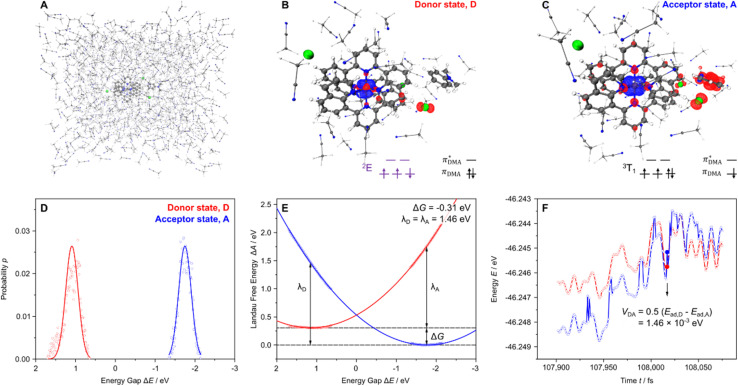
(A) AIMD setup with one [Cr(dqp)_2_]^3+^ complex, one DMA molecule, three chloride ions and 31 AcN solvent molecules in the QM box (30 × 22 × 22 Å^3^), embedded in a MM box (59.7 × 47.7 × 52.1 Å^3^), which contains further 729 solvent molecules. (B) and (C) Spin density of QM region within the donor and the acceptor state respectively; blue and red represents α and β-spin, respectively. (D) Probability (dots) of the various energy gaps Δ*E* of the donor (red) and the acceptor state (blue) states along the trajectory as well as ideal Gaussian distributions; bin size is 1 kJ mol^−1^. (E) Fitted Marcus parabolas of the donor and acceptor states using the Warshel histogram approach; driving force and reorganization energies are indicated. (F) Intersection region of the diabatic potential energy curves along the trajectory and electronic coupling obtained by the minimum energy splitting method.

In order to investigate the ET kinetics between an excited [Cr(dqp)_2_]^3+^ (^2^E or ^2^T_1_ states) and DMA in AcN, we rely on *ab initio* molecular dynamics in combination with a QM/MM (quantum mechanical/molecular mechanical) approach. Thereby, the reactants – one [Cr(dqp)_2_]^3+^ complex as well as one DMA molecule – as well as the first solvent shell (31 AcN molecules) and respective counter ions are described quantum mechanically within a 30 × 22 × 22 Å^3^ box using density functional theory (BLYP with DZVP-MOLOPT-SR-GTH basis sets and D3BJ dispersion correction).^55,57,72–74^ As visualized in Fig. 6A, this QM system is embedded in a MM box of 59.7 × 47.7 × 52.1 Å^3^ containing additional 729 solvent molecules as described using the GAFF2 force field (*T* = 100 K).^75–78^ Further details with respect to the computational setup are summarized in the SI.

Based on this setup, the reductive quenching of the excited Cr(iii) complex by DMA was modelled within the semi-classical Marcus picture of ET. Within Marcus theory,^79–81^ electron transfer processes are described based on diabatic potential energy curves (PECs) from a donor (D) to an acceptor state (A), while rare thermal fluctuations of the bath, *e.g.* the solvent environment, lead to structural changes of the reactants which may result in electron transfer in the vicinity of the crossing between the two diabatic PECs. This way, the rate constant for a electron transfer process (*k*_ET_) at a given temperature (*T*) is provided by the driving force or Gibbs free energy (Δ*G*_0_) and the reorganization energy (*λ*; comprising inner and outer sphere contributions). Furthermore, the electronic communication between the donor and the acceptor state is given by the potential coupling between the two diabatic states (*V*_DA_). Further information regarding the utilized semi-classical Marcus picture are provided in the computational details of the SI (section “Intermolecular Electron Transfer” and in particular eqn (11)). Previously, this methodology was utilized and evaluated against (dissipative) quantum dynamical simulations within our group to model light-driven intramolecular electron transfer events in various transition metal complexes^82–84^ and photocatalysts^85–87^ as well as in the context of redox-active organic batteries.^88,89^

In the present contribution, we extend our computational approach to intermolecular ET reactions, while the electron donor state is defined by [Cr(dqp)_2_]^3+^ within its lowest energy doublet state in combination with a non-oxidized DMA, see Fig. 6B. Surprisingly, constrained DFT reveals this lowest energy doublet state of the chromium(iii) complex as the true spin-flip state (^2^E; and not a ^2^T_1_ state) without the need of applying further, *e.g.*, broken symmetry approaches. In contrast, the acceptor state comprises of a singly reduced chromium complex and a singly oxidized DMA molecule, see Fig. 6C. Within the acceptor state, a Cr(ii) complex is predicted, which features the additional electron within one t_2g_ orbital, which leads consequently to a triplet configuration of the d^4^ system (^3^T_1_). Notably, partial spin density is also observed in low-lying 
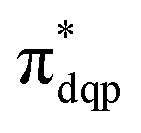
 orbitals of the coordinating dqp ligands. Thus, the reduction event involves mainly the coordination center and the ligand sphere to a lesser extent. Experimentally, the first reduction event in [Cr(dqp)_2_]^3+^ was assigned to a ligand-based process.^37^ However, there is a constant debate^90,91^ regarding the redox chemistry of Cr(iii) complexes and the localization of the respective redox events to be either metal-based or ligand-based, which is reflected by the present computational results which associate this reduction to both metal and ligand contributions. In contrast, the electron hole is clearly localized in the formally doubly occupied highest π_DMA_ orbital which combines contributions from its phenyl ring and the lone-pair of the amino group. In addition, and less prominently, the chloride ions (*p*_Cl_) also participate as partial donors, see Fig. 6C.

Based the donor and acceptor states (Fig. 6B and C), the potential energy landscape was sampled by means of AIMD simulations in the vicinity of their previously relaxed equilibrium structures. Thereby, the relaxed donor structure represents the encounter complex involving the photoexcited Cr(iii) complex and the DMA donor in explicit solvent environment. Structurally, this encounter complex is characterized by a preorientation of the DMA molecule along the aromatic plane of one dqp ligand in an almost linear configuration. Notably, the amino group of DMA is pointing away from the metal center, while the shortest interatomic distance of merely 1.84 Å is predicted between the donor and the acceptor molecules or *d*_Cr–N_ = 12.53 Å between the chromium atom and the (amino)-nitrogen of DMA. Within the acceptor state geometry, a slight rotation of the oxidized DMA radical (DMA^+^˙) with respect to the Cr complex is observed. In consequence, the distance between the chromium atom and the amino group (N atom) – mainly involved in the oxidation – decreases slightly to ∼11.7 Å due to electrostatic effects, while the shortest interatomic distance remains with 2.19 Å almost unchanged. All structural properties and videos based on the AIMD runs are available *via* Zenodo.^92^

During the data processing and the construction of the Marcus parabolas, we followed the histogram approach proposed by Warshel,^93,94^ which defines the vertical energy gap (Δ*E*) between the donor and the acceptor state as the reaction coordinate. Both probability distributions of the energy gap (Fig. 6D), *i.e.* for the donor and the acceptor equilibria, follow almost ideal Gaussian distributions. Subsequently, we improved the accuracy of the Marcus parabola fitting by using the linear response approximation, see eqn (9) the SI.^94–98^ This allows for the generation of twice as many points on the two free energy curves without performing additional simulations, see Fig. 6E. Consequently, two identical reorganization energies *λ*_D_ = *λ*_A_ = 1.46 eV as well as a negative driving force Δ*G*_0_ = −0.31 eV were obtained. Furthermore, the electronic communication as rationalized by means of the potential coupling *V*_DA_ = 1.46 × 10^−3^ eV for the (diabatic) donor-acceptor pair was obtained by the energy splitting method.^82,83,85,87^ The coupling was obtained based on the switching of states along the AIMD run (Fig. 6F). Finally, the intermolecular ET rate was calculated *via* semi-classical Marcus theory; detailed information regarding the data processing and the conceptional background are provided in the SI, *i.e.* eqn (11). Based on the low temperature as utilized in the AIMD simulations (*T* = 100 K) a very slow intermolecular ET rate of 1.97 × 10^−1^ s^−1^ is obtained. Experimentally, the bimolecular ET kinetics between the excited [Cr(dqp)_2_]^3+^ complex and the DMA donor were studied *via* electrochemical investigations as well as luminescence experiments and subsequent Stern–Volmer analysis. Based on the experimental driving force of approximately −0.41 eV, a quenching rate of 8.3 × 10^9^ M^−1^ s^−1^ was obtained at room temperature (293 K) in acetonitrile for the pseudo-first-order reaction.^36^

Thus, our DFT-predicted driving force −0.31 eV (at *T* = 100 K) is in good agreement with the electrochemically obtained value of −0.41 eV. Therefore, we recalculated our ET rate at a temperature of 293 K and based on the DMA concentration within our periodic box (59.7 × 47.7 × 52.1 Å^3^, see Fig. 6A), which yields a pseudo-first-order rate constant of 3.41 × 10^8^ M^−1^ s^−1^. Notably, the driving force is formally temperature-dependent. However, only slight structural changes are predicted between the equilibrated donor and acceptor state (Fig. 6B and C). Thus, the impact of the entropic term and, therefore, temperature-dependency of the Δ*G* is expected to be negligible. Therefore, both the computational modelling and the experimental findings reveal efficient light-induced reductive quenching of the Cr(iii) chromophore by DMA while the ET is fast with respect to the excited state lifetime of [Cr(dqp)_2_]^3+^. The difference between the theoretical and the Stern–Volmer-based ET rate of approximately one order of magnitude is mainly a consequence of the intrinsic challenges of density functional theory to describe the complicated electronic structure of [Cr(dqp)_2_]^3+^ and especially its low-lying doublet excited states, *i.e.*^2^E *vs.*^2^T_1_, correctly, which leads to an underestimation of the driving force by 0.1 eV. Furthermore, the counter ion was changed from PF_6_^−^ to the smaller Cl^−^ in the computational modelling.

## 4 Conclusions

The present computational study carefully evaluates the excited states involved in spin-flip luminescence of a Cr(iii) complex, *i.e.*[Cr(dqp)_2_]^3+^, as well as its photoredox chemistry in combination with a reductive quencher as previously investigated experimentally.^36^ The present computational contribution allows one to rationalize the intramolecular light-driven processes associated with spin-flip phenomena in d^3^-based transition metal complexes as well as utilizing these millisecond-lived excited states in photoredox chemistry. Such findings are of significance for the development of efficient noble-metal free photocatalysts.

The excited-state landscape at the Franck–Condon point was carefully assessed by state-of-the-art multiconfigurational simulations which account for both static and dynamic electron correlation. These MS-RASPT2//RASSCF results were utilized to evaluate the performance of cost-efficient TDDFT simulations to describe the complex electronic structure of such a molecular ruby and the respective light-driven processes, *i.e.*, associated with its ligand field based transitions (^4^A_2_ → ^4^T_2_ and ^4^A_2_ → ^4^T_1_). Subsequently, our computational focus was set on evaluating the spin-flip phenomenon and the underlying thermodynamics as well as the kinetics of various intersystem crossing pathways, which lead eventually to the population of millisecond-lived doublet states. Based on the present MS-RASPT2 results, ultrafast ISC leads within sub-ps to few picoseconds to an almost identical population of the quasi-isoenergetic ^2^E and ^2^T_1_ states (Fig. 4). Finally, the emission of the present molecular ruby is mostly associated with the slightly shorter-lived ^2^E states.

Subsequently, the focus of our computational investigation was set beyond the description of the photophysical properties and the mechanistic understanding of the spin-flip phenomena in Cr(iii) complexes, namely on harvesting these extraordinarily long-lived excited states in photoredox processes. To this aim, *ab initio* molecular dynamics simulations were carried out to investigate the photoinduced reductive quenching of an excited [Cr(dqp)_2_]^3+^, by an organic donor, *i.e. N*,*N*-dimethylaniline (DMA),^36^ as investigated previously based on electrochemical and luminescence and transient absorption experiments as well as subsequent Stern–Volmer analysis. These simulations allowed the structural configuration of the encounter complex (in acetonitrile solution) to be assessed and for the electronic structure of the redox intermediates associated with the intermolecular electron transfer to be elucidated. Based on our simulations the reduction of the chromium complexes is mostly metal-based and leads to a d^4^ triplet configuration, while only a minor electron density contribution is transferred from the highest energy π_DMA_ orbital the π* orbitals of the dqp ligands. Finally, the thermodynamics and kinetics of the light-driven intermolecular electron transfer were investigated within the semi-classical Marcus picture. Based on our computational modelling, a driving force of −0.31 eV and a reorganization energy 1.46 eV – which involves both inner and outer sphere contributions – was predicted, which is in good agreement with electrochemical results. Based on these results an ET rate of 3.41 × 10^8^ M^−1^ s^−1^ was obtained for the pseudo-first order excited state quenching reaction, which is particularly fast for typically weakly coupled intermolecular transfer processes. This rate constant as rationalized by AIMD simulations is in good agreement with the experimental Stern–Volmer kinetics, which predict an even slightly faster reductive quenching of the excited chromium complex.

Therefore, the insights at the molecular level presented herein will contribute to the transformation from noble metal-based photocatalysis to efficient noble metal-free photocatalysts.

## Author contributions

The project was supervised and coordinated by S. Kupfer, in consultation with O. Wenger. G. Yang performed all computational simulations and was the primary contributor to writing the manuscript, aided by S. Kupfer and G. Shillito. P. Seeber guided the setup and analysis of the AIMD simulations. All authors contributed to editing of the manuscript.

## Conflicts of interest

There are no conflicts to declare.

## Supplementary Material

SC-016-D5SC05170C-s001

## Data Availability

All optimized structures, high resolution images and trajectories are available *via* the free online repository Zenodo (DOI: https://doi.org/10.5281/zenodo.15849517).^92^ The data supporting this article have been included in the main manuscript as well as in the SI. Supplementary information: computational details (geometry optimization, ground and excited state properties within the Franck–Condon region, setup of multiconfigurational simulations, intermolecular electron transfer kinetics and AIMD setup), simulated UV-vis spectra, Marcus parabolas, excited state properties and composition. See DOI: https://doi.org/10.1039/d5sc05170c.
