# Supplementary material for: Unraveling the photoredox chemistry of a molecular ruby
Source: Chem Sci. 2025 Sep 10;16(39):18113–25. doi: 10.1039/d5sc05170c (PMC12459676; doi:10.1039/d5sc05170c)
Supplement: SC-016-D5SC05170C-s001 [file SC-016-D5SC05170C-s001.pdf]

Supplementary Information (SI) for Chemical Science.
This journal is © The Royal Society of Chemistry 2025

Supporting Information

Unraveling the Photoredox Chemistry of a Molecular Ruby

Guangjun Yang,[a] Georgina E. Shillito,[a] Phillip Seeber,[a,b] Oliver S. Wenger[c] and Stephan Kupfer[a]*

[a] Institute of Physical Chemistry, Friedrich Schiller University Jena,

Lessing Straße 4, 07743 Jena, Germany

[b] University Computer Centre, Friedrich Schiller University Jena,

Am Johannesfriedhof 2, 07743 Jena, Germany

[c] Department of Chemistry, University of Basel, St. Johanns-Ring 19,

4056 Basel, Switzerland

E-mail: stephan.kupfer@uni-jena.de

Computaional Details

Photophysical Properties of [Cr(dqp)$_2$]$^{3+}$

All ground state calculations, for the Cr(III) complex **[Cr(dqp)$_2$]$^{3+}$**, were performed utilizing the Gaussian 16 Rev. C.02 program[1]. The quartet ground state equilibrium structure and electronic properties with application of D_2 symmetry, were obtained at the density functional level of theory (DFT) using the B3LYP[2-7] exchange correlation (XC) functional with the def2-SVP[8, 9] basis set as well as the respective core potentials. Subsequently, a vibrational analysis was carried out for optimized ground state structure to verify that a local minimum on the 3*N*-6 dimensional potential energy (hyper-)surface was reached. Effects of interaction with the acetonitrile solvent (CH_3CN: ε = 36.688, *n* = 1.807) were taken into account by the solute electron density (SMD) variant of the integral equation formalism of the polarizable continuum model (IEFPCM).[10, 11] All calculations were performed including Grimme's D3 dispersion correction with Becke-Johnson damping (D3BJ).[12] Subsequently, aiming to provide insight into the exited-state properties, *i.e.* excitation energies, oscillator strengths, transition dipole moments and electronic characters of the 100 lowest excited quartet and doublet states within the Franck-Condon region, time dependent DFT (TDDFT) calculations were performed using the correlation-consistent polarized valence double-zeta (cc-pVDZ) basis set[13-15] and the same XC functional as mentioned above in the preceding optimization calculation.

However, static correlation stemming from near-degenerate electronic configurations (Slater determinants) is insufficiently treated by TDDFT, thus the complete active space self-consistent field (CASSCF) methodology from the family of *ab initio* multiconfigurational methods was initially considered. However, while multiconfigurational calculations allow to provide an unbiased description of the photophysics, the computational demand raises quickly with the size of the active space (AS). An appropriate AS for **[Cr(dqp)$_2$]$^{3+}$** would be Cr-centered, comprising seven electrons in seven molecular orbitals, (7,7), a (52,52) with the π_{tpy}/π_{tpy}^* system

of dqp ligands, as well as a (0,5) containing five empty 4*d* orbitals for considering double *d* shell effects. Consequently, an AS (59,64) is obtained, which is computationally prohibitive without further constrain. To restrict the number of configuration state functions (CSFs) in the CASSCF methodology, the restricted active space (RAS)SCF method[16-18] was introduced, which allows the computational demand to be reduced by splitting the AS into three subspaces. RAS1 holds mostly doubly occupied orbitals with a predefined number of maximal electron holes, while the RAS3 subspace contains mostly unoccupied orbitals where a defined number of electrons is allowed to be excited into, the RAS2 subspace plays the same role as the AS in CASSCF methodology where a full configuration interaction calculation is performed. In order to label the RASSCF calculations, the notation RAS (*n*,*l*,*m*;*i*,*j*,*k*) of Gagliardi and co-workers[19] was introduced. The index *n* denotes the total number of active electrons, *l* represents the maximum permitted number of holes in the RAS1, while *m* is the maximum number of allowed electrons in the RAS3. The labels *i*, *j*, and *k* refer to the number of active orbitals in RAS1, RAS2, and RAS3, respectively. All multiconfigurational calculations were performed in MOLCAS 8.4[20] using the geometry obtained from Gaussian ground state optimization with B3LYP XC functional. In addition, the cc-pVDZ basis set was applied with the (second-order) Douglas-Kroll (DK) Hamiltonian[21, 22], which is utilized for considering relativistic effects. In order to obtain spin-orbit coupling, the restricted active space state interaction (RASSI) method was utilized computing interaction Hamiltonians between different RASSCF wave functions (representing electronic states). Finally, the restricted active space self-consistent field with second-order perturbation (RASPT2) method was employed to address the dynamic correlation, which is induced by instantaneously mutual repulsion of electrons. When states within the first-order interacting space approach near-degeneracy, known as intruder states, the second-order correction energy becomes infinite. To avoid this fraction, a level shift 0.3 was introduced.

In order to reduce computational cost, the strategy we adopted is first to apply D_2 symmetry in these calculations, following by a rational distribution of active electrons

and orbitals among three subspaces. Specifically, the (7,7) of the Cr center is included in RAS2. These seven orbitals comprise of two pairs of σ/σ* orbitals, reflecting the linear combinations of the $3d_{x^2-y^2}$ and $3d_{z^2}$ lone-pairs of the ligands of suitable symmetry, as well as the $3d_{xy}$, $3d_{xz}$ and $3d_{yz}$ orbitals of the chromium atom. Furthermore, in order to better describe states with greater involvement of the ligands, the lowest four unoccupied π* orbitals of the dqp ligands, distributed in each four D_2 symmetry point group (a, b_1, b_2, and b_3), were included in RAS2. To further improve the description of these π* orbitals, the corresponding π orbitals in each symmetry point group were assigned to RAS1 subspace. Finally, five empty 4*d* orbitals of chromium were allocated into RAS3 for inclusion of double d shell effects. Consequently, the RASs (15,2,2;4,11,5) was constructed which comprises more than nine million configuration state functions, covering the lowest eight quartet and the lowest eight doublet roots of **$[Cr(dqp)_2]^{3+}$**.

Subsequently, to quantify the rate of intersystem crossing (ISC), the simplified formula of rate constant with Fourier transformed Lorentzian dephasing[23] was utilized. For the energy downhill $Q_i \rightarrow D_j$ rate constant, the formula is shown as below

$$k_{ISC\downarrow} = \frac{2\gamma}{\hbar(\Delta E_{ij}^2 + \gamma^2)} |SOC|^2$$

Eq. 1

where the γ represents the half width at half maximum (HWHM) of the simulated UV-vis spectrum, ΔE_{ij} is the vertical energy difference at FC region, SOC denotes the spin-orbit coupling term and ħ is the reduced Planck constant. In a similar manner, when the $Q_i \rightarrow D_j$ non-radiative transition is an energy-uphill transition, the correction is added:

$$k_{ISC\uparrow} = \frac{2\gamma}{\hbar(\Delta E_{ij}^2 + \gamma^2)} |SOC|^2 exp\left(- \frac{\Delta E_{ij}}{k_B T} \right)$$

Eq. 2

where k_B is the Boltzmann constant and T is the temperature (T = 293.15 K).

The phosphorescence rate k_p for a transition from the doublet state $|i\rangle$ to the quartet ground state $|f\rangle$, assuming photon emission over all directions and polarizations, and after a rotational average over randomly oriented molecules, is given by the Einstein A_{if} coefficient:[24]

$$k_P = A_{if} = \frac{\Delta E_{ij}^3}{3\varepsilon_0 \pi c^3 \hbar^4} |\vec{\mu}_{if}|^2 \qquad \text{Eq. 3}$$

in which $\Delta E_{if} = (E_i - E_f)$ is the Bohr pulsation between the ith excited state and ground state f, and $\vec{\mu}_{if}$ is the transition dipole moment. ε_0, c and $\hbar$ are the vacuum permittivity, speed of light in vacuum and the Planck's constant, respectively.

Intermolecular Electron Transfer

To reduce the computational demand of quantum mechanical and molecular mechanical (QM/MM) simulations, we simplify the **[Cr(dqp)$_2$](PF$_6$)$_3$** model with the replacement of PF_6^- anions to Cl^- anions. Thus, the QM/MM system consists of a **[Cr(dqp)$_2$]Cl$_3$** complex as well as a single N,N-Dimethylaniline (DMA) molecule in a 760-acetonitrile (CH_3CN) molecule environment. A cubic QM/MM box with three dimension of 59.7 x 47.7 x 52.1 Å^3 was initially constructed using Packmol 20.13.0[25]. The **[Cr(dqp)$_2$]Cl$_3$** complex, DMA, and surrounding 31 CH_3CN molecules are treated as the QM region in a 30 × 22 × 22 Å^3 cell, while the other CH_3CN molecules are included within the MM region. The MM region is described using the GAFF2 force field[26-29] by Amber 21 software[30]. All software packages utilized for the QM/MM simulations were provided via NixOS-Qchem.[31]

Ab initio molecular dynamics (AIMD) simulations were performed using CP2K 2024.1 software[32, 33] in the framework of fully periodic hybrid Gaussian and plane wave DFT. The BLYP functional[3, 5, 34]was employed in combination with polarized short-range double ζ valence basis sets (DZVP-MOLOPT-SR-GTH)[35] for the Cr atom, while DZVP-MOLOPT-GTH basis set was used for the other atoms. Notably, hybrid functionals such as the B3LYP functional as utilized in the previously outlined electronic structure simulations of **[Cr(dqp)$_2$]$^{3+}$** are computational too demanding for the present AIMD simulations. However, and in contrast to their insufficient description of excited state properties in coordination compounds, GGA functionals, such as BLYP are capable of sufficiently describing their electronic structure within electronic ground state as well as atomization energies (*i.e.* of doublet ground state in the present scenario).[36, 37] Furthermore, GTH-BLYP pseudopotentials[38-40] were applied. The simulations used a plane wave cutoff of 850 Ry and a relative cutoff of 60 Ry on five real-space grids. To account for long-range interactions, *i.e.* dispersion, Grimme's D3BJ damping was employed. The MM system was equilibrated for 7 ps with a time-step of 0.5 fs in a canonical (*NpT*) ensemble at T = 100 K, enforced by the canonical sampling through velocity rescaling (CSVR) thermostat (τ = 50 fs) proposed by Bussi[41]. Subsequently,

the equilibrated QM system of acceptor (A) state was optimized for 74 ps using the same setup as described above. Besides, the equilibrated QM system of donor (A) state was optimized starting from A state equilibrium for 20 ps using the constrained DFT (CDFT) calculation. The CDFT constrains the DMA valence electron numbers as 48 making the whole molecule neutral. Figure S1 indicates the energy curves remain stable in the displayed area, thereby both D and A states reach their equilibria.

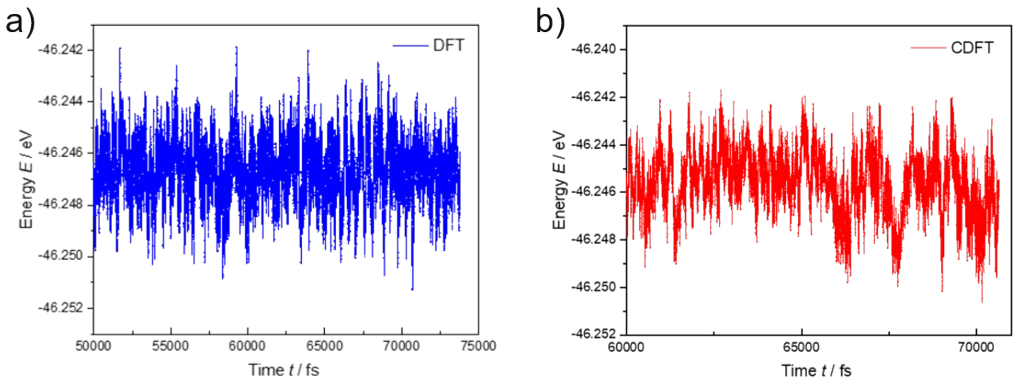

Figure S1. a) DFT optimization b) CDFT optimization energy curves, indicating both donor and acceptor states reach their equilibria.

On the one hand, we sampled 5000 snap shots every 0.5 fs near equilibrium of A state for obtaining A state energy $E_A(R_A)$. For each single point, a CDFT calculation was carried out for obtaining D state energy $E_D(R_A)$. On the other hand, another 5000 snap shots were sampled near D state equilibrium within CDFT calculations for obtaining D state energy $E_D(R_D)$. In the subsequent CDFT calculations, the DMA valence electrons was constrained to 47 in order to calculate A state energy $E_A(R_D)$. R_D and R_A denote the reaction coordinate of D and A states, respectively. $\Delta E(R_D)$ and $\Delta E(R_A)$ are the vertical energy gap of D and A states along two coordinates, which can be expressed as below,

$$\Delta E(R_A) = E_A(R_A) - E_D(R_A) \quad \text{Eq. 4}$$

$$\Delta E(R_D) = E_A(R_D) - E_D(R_D)$$

If donor and acceptor are well separated, the kinetics of ET can be described in the nonadiabatic limit. In this regime, reactant and product are described by two (Landau)

free-energy profile curves A_M (M = D or A) along a reaction coordinate, which is taken to be the energy gap equation,

$$A_M(\Delta E) = -k_B T ln p_M(\Delta E) + G_M \quad \text{Eq. 5}$$

$-k_B T ln p_M(\Delta E)$ is the diabatic free energy of D and A states, denoted as G_M. The free energy curve is proportional to the logarithm of the probability p of ΔE, $p_M = \langle \delta(\Delta E_M - \Delta E) \rangle_M$, where δ is the Dirac delta function. ΔE currently is a series of integer values that are grouped into specific intervals, referred to as the bin size. G_M is the constant and follows $G_A - G_D = \Delta G_0$, where ΔG_0 denotes driving force. Applications have shown that the gap energy is indeed a good reaction coordinate for pure ET reactions.[42, 43] The driving force ΔG_0 for nonadiabatic pure ET is then obtained from the half sum of the expectation energy of D and A states, shown as

$$\Delta G_0 = (\langle \Delta E \rangle_D + \langle \Delta E \rangle_A)/2 \quad \text{Eq. 6}$$

The reorganization free energy, which quantifies the energetic cost of distorting the system from the equilibrium configuration of one diabatic state to that of the other, while staying on the same diabatic potential energy curve, is given by

$$\lambda_D = A_D\left(\Delta E_A^{min}\right) - A_D\left(\Delta E_D^{min}\right) \quad \text{Eq. 7}$$

$$\lambda_A = A_A\left(\Delta E_D^{min}\right) - A_A\left(\Delta E_A^{min}\right)$$

Or estimated by

$$\lambda_D = \lambda_A = \left|\langle \Delta E \rangle_D + \langle \Delta E \rangle_A\right|/2 \quad \text{Eq. 8}$$

Eq.6 and Eq.8 can be derived by the linear response[42-46] between the energy gap ΔE and Landau free energy difference:

$$A_A(\Delta E) - A_D(\Delta E) = \Delta E \quad \text{Eq. 9}$$

The diabatic free energy G_M can be written in another form:

$$G_M = \frac{K_M}{2}(\Delta E - \Delta E_M)^2 + \frac{k_B T}{2} ln \frac{2\pi k_B T}{K_M} \qquad \text{Eq. 10}$$

In order to be compatible with Eq.9, the coefficients of the quadratic term in Eq. 10 must be equal, which means that the D and A state curvatures are the identical ($K_D = K_A = K$). Substituting K in Eq. 9, the Eq. 6 and Eq. 8 can be obtained by forcing the coefficients of the first-order term to 1 and removing the zeroth order term. There is an alternative route to derive by rewriting the Zwanzig relation in the form of cumulant expansions.[47]

Based on this setup, the reductive quenching of the excited Cr(III) complex by DMA was modelled within the semi-classical Marcus picture of ET. Within Marcus theory, electron transfer processes are described based on diabatic potential energy curves (PECs) from a donor (D) to an acceptor state (A), while rare thermal fluctuations of the bath, e.g. the solvent environment, lead to structural changes of the reactants which may result in electron transfer in vicinity of the crossing between the two diabatic PECs. This way, a rate constant for the electron transfer process (k_{ET}) is given by:[48-50]

$$k_{ET} = \frac{2\pi}{\hbar}|V_{DA}|^2(4\pi\lambda k_B T)^{-\frac{1}{2}}\exp\left(-\frac{(\Delta G_0 + \lambda)^2}{4\lambda k_B T}\right), \qquad \text{Eq. 11}$$

where ΔG_0 is the driving force or Gibbs free energy, λ the reorganization energy (comprising inner and outer sphere contributions), furthermore, T is the temperature and k_B is the Boltzmann constant. The electronic communication between the donor and the acceptor state is given by the potential coupling between the two diabatic states (V_{DA}). Previously, this methodology was utilized and evaluated against (dissipative) quantum dynamical simulations within our group to model light-driven intramolecular electron transfer events various transition metal complexes[51-53] and photocatalysts[54-56] as well as in the context of redox-active organic batteries.[57, 58]

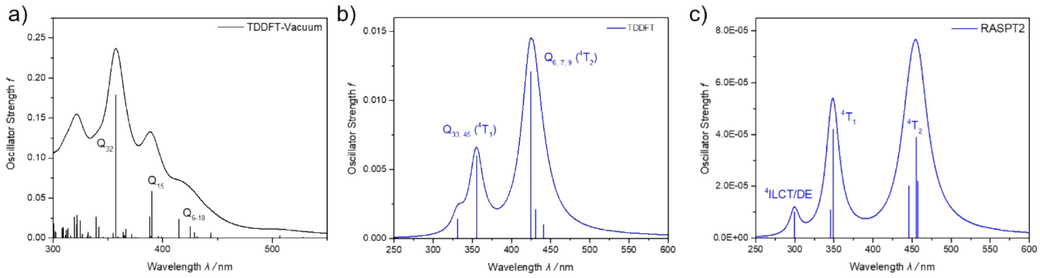

Figure S2. a) Simulated UV-vis absorption spectra of **[Cr(dqp)$_2$]$^{3+}$** as predicted at the at the TDDFT level of theory in vacuum. b) The TDDFT-simulated UV-vis absorption spectrum in vacuum. MC/mixed transitions are exclusive selected. c) Simulated UV-vis absorption spectrum in RASPT2 level of theory. Simulated transitions are broadened by Lorentzian functions with a full width at half maximum of 0.1 eV.

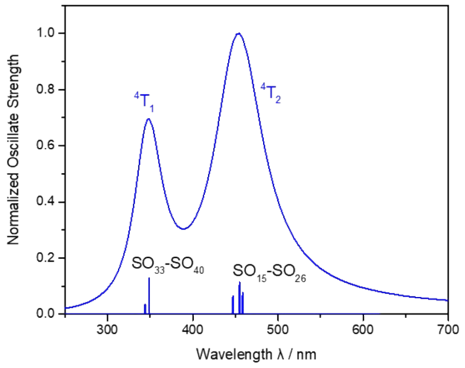

Figure S3. Simulated spin-orbit UV-vis absorption spectrum of **[Cr(dqp)$_2$]$^{3+}$** at RASPT2 level of theory in Molcas 8.4.

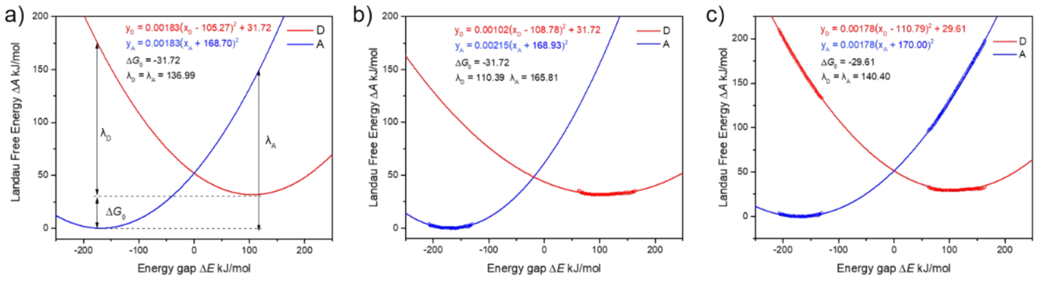

Figure S4. a) Ideal Marcus parabolas when $\Delta G_0 = (\langle \Delta E \rangle_D + \langle \Delta E \rangle_A)/2$ and $\lambda_D = \lambda_A = |\langle \Delta E \rangle_D + \langle \Delta E \rangle_A|/2$. b) Marcus parabolas for ET between **[Cr(dqp)$_2$]$^{3+}$** and DMA molecule using the original low-energy data near equilibrium of the states. c) Marcus parabolas for ET between **[Cr(dqp)$_2$]$^{3+}$** and DMA molecule using both the original low-energy data and high-energy data. The high-energy data is obtained based on linear response approximation; bin size is 1 kJ/mol.

Table S1. The N-Cr-N angles of a **$[Cr(dqp)_2]^{3+}$,** exhibiting a deviation of only 1–3° from the perfectly octahedral geometry.

Character	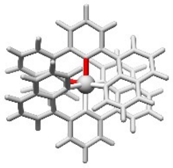	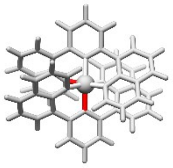
Angle	88.55	91.45
Character	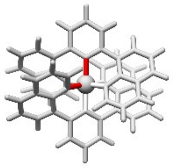	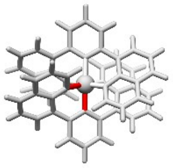
Angle	91.45	88.55
Character	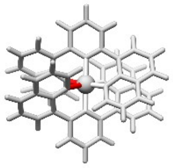	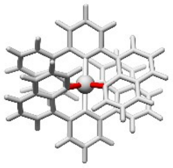
Angle	93.30	86.77

Table S2. Simulated excited state properties of the low-lying bright quartet excited states of **$[Cr(dqp)_2]^{3+}$** gas phase such as excitation energies (in eV), excitation wave lengths (in nm), oscillator strengths, spin contamination, MO pairs, leading transitions as represented by charge density differences (CDDs; charge transfer takes place from red to blue). All results were obtained using the B3LYP functional as implemented in Gaussian 16.

Transition ($^4A_{2g} \rightarrow Q_x$)	ΔE / eV	λ / nm	f	$\langle s^2 \rangle$	Character
Q_6	2.81	442	0.001	3.90	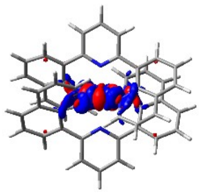
Q_7	2.88	431	0.0021	3.98	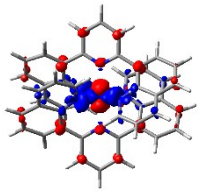
Q_8	2.89	429	0.0071	4.19	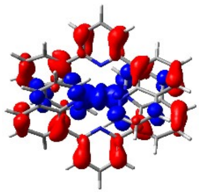
Q_9	2.92	425	0.0121	4.18	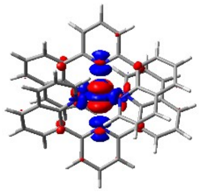
Q_{10}	2.99	415	0.0236	4.35	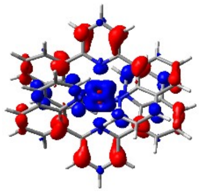
Q_{15}	3.18	390	0.0583	4.73	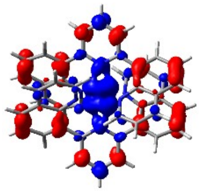

Transition ($^4A_{2g} \rightarrow Q_x$)	ΔE / eV	λ / nm	f	⟨ s^2 ⟩	Character
Q_{32}	3.47	357	0.1788	4.23	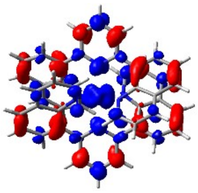
Q_{33}	3.49	355	0.0060	4.33	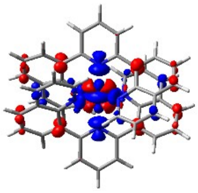
Q_{45}	3.74	331	0.0014	4.30	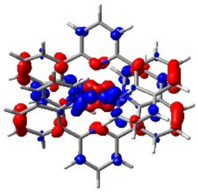
Q_{46}	3.75	330	0.0000	4.24	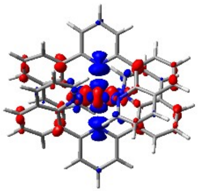

Table S3. Simulated excited state properties of the low-lying bright quartet excited states of **$[Cr(dqp)_2]^{3+}$** in an acetonitrile (AcN) model such as excitation energies (in eV), excitation wave lengths (in nm), oscillator strengths, spin contamination, MO pairs, leading transitions as represented by charge density differences (CDDs; charge transfer takes place from red to blue). All results were obtained using the B3LYP functional as implemented in Gaussian 16.

Transition ($^4A_{2g} \rightarrow Q_x$)	ΔE / eV	λ / nm	f	⟨s²⟩	Character
Q_5	2.82	440	0.0083	4.26	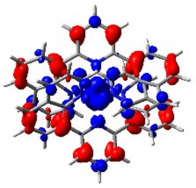
Q_6	2.85	435	0.0004	3.93	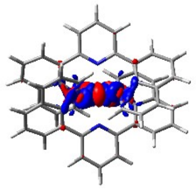
Q_7	2.91	426	0.0019	3.96	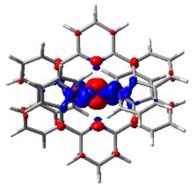
Q_8	2.91	425	0.0097	4.15	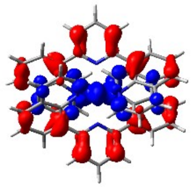
Q_9	2.95	420	0.0386	4.21	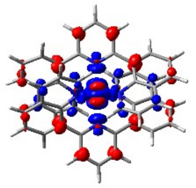
Q_{10}	3.01	412	0.0184	4.14	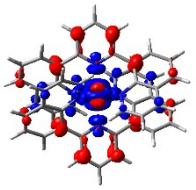

Transition ($^4A_{2g} \rightarrow Q_x$)	ΔE / eV	λ / nm	f	$\langle s^2 \rangle$	Character
Q_{15}	3.18	390	0.1274	4.09	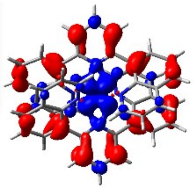
Q_{32}	3.48	356	0.2505	4.26	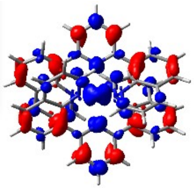
Q_{33}	3.53	351	0.0062	4.26	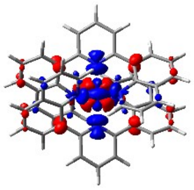
Q_{45}	3.77	328	0.0000	4.25	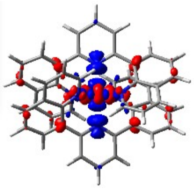
Q_{46}	3.78	328	0.0019	4.30	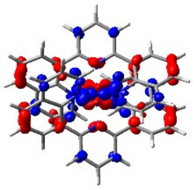

Table S4. Simulated ground and low-lying bright quartet excited states properties of of **$[Cr(dqp)_2]^{3+}$** such as excitation energies (in eV), oscillator strengths, spin contamination, leading transitions as represented by the occupancy of electrons in molecular orbitals. (charge transfer takes place from red to blue, green denotes a spin flip). u and d represent α and β spin electron, respectively. All results were obtained at the RASPT2 level of theory as implemented in Molcas 8.4.

Ex.	ΔE / eV	Os.	Transition Character																				Wt. /%
			RAS1				RAS2											RAS3					
			π_a	π_{b2}	π_{b1}	π_{b3}	$\sigma_{3d_{z^2}}$	$3d_{xy}$	$\sigma^*_{3d_{z^2}}$	π^*_a	$3d_{xz}$	π^*_{b2}	$\sigma_{3d_{x^2-y^2}}$	$\sigma^*_{3d_{x^2-y^2}}$	π^*_{b1}	$3d_{yz}$	π^*_{b3}	$4d_{xy}$	$4d_{xz}$	$4d_{yz}$	$4d_{x^2-y^2}$	$4d_{z^2}$	
$^4A_2$	0.00	-	2	2	2	2	2	u	0	0	u	0	2	0	0	u	0	0	0	0	0	0	86
$^4T_2$	2.71	2.16E-5	2	2	2	2	2	0	0	0	u	0	2	u	0	u	0	0	0	0	0	0	85
$^4T_2$	2.72	3.87E-5	2	2	2	2	2	u	u	0	u	0	2	0	0	0	0	0	0	0	0	0	66
			2	2	2	2	2	u	0	0	0	0	2	u	0	u	0	0	0	0	0	0	20
$^4T_2$	2.78	2.02E-5	2	2	2	2	2	u	0	0	u	0	2	u	0	0	0	0	0	0	0	0	51
			2	2	2	2	2	u	u	0	0	0	2	0	0	u	0	0	0	0	0	0	35
$^4T_1$	3.55	4.24E-5	2	2	2	2	2	u	u	0	u	0	2	0	0	0	0	0	0	0	0	0	20
			2	2	2	2	2	u	0	0	0	0	2	u	0	u	0	0	0	0	0	0	67
$^4T_1$	3.59	1.10E-5	2	2	2	2	2	u	0	0	u	0	2	u	0	0	0	0	0	0	0	0	36
			2	2	2	2	2	u	u	0	0	0	2	0	0	u	0	0	0	0	0	0	50
$^4T_1$	3.83	0.00	2	2	2	2	2	0	u	0	u	0	2	0	0	u	0	0	0	0	0	0	79
^{4}ILCT/ ^{4}DE	4.14	1.00E-5	2	2	u	2	2	u	0	d	u	0	2	0	0	u	0	0	0	0	0	0	11
			2	2	d	2	2	u	0	u	u	0	2	0	0	u	0	0	0	0	0	0	8
			2	2	2	u	2	d	0	0	u	0	2	0	0	u	u	0	0	0	0	0	7
			2	u	2	2	2	d	0	0	u	0	2	0	0	u	u	0	0	0	0	0	10

			u	2	2	2	2	d	0	0	u	0	2	0	u	u	u	0	0	0	0	0	8
			2	u	2	2	2	u	0	0	u	0	2	0	0	u	d	0	0	0	0	0	9

Table S5. Simulated excited state properties of the low-lying doublet excited states of **[Cr(dqp)$_2$]$^{3+}$** such as excitation energies (in eV), oscillator strengths, spin contamination, leading transitions as represented by the occupancy of electrons in molecular orbitals. (charge transfer takes place from red to blue, green denotes a spin flip). u and d represent α and β spin electron, respectively. All results were obtained at the RASPT2 level of theory as implemented in Molcas 8.4.

Ex.	Δ*E* / eV	Os.	Transition Character																				Wt. /%
			RAS1				RAS2											RAS3					
			π_a	π_{b2}	π_{b1}	π_{b3}	$\sigma_{3d_{z^2}}$	$3d_{xy}$	$\sigma^*_{3d_{z^2}}$	π^*_a	$3d_{xz}$	π^*_{b2}	$\sigma_{3d_{x^2-y^2}}$	$\sigma^*_{3d_{x^2-y^2}}$	π^*_{b1}	$3d_{yz}$	π^*_{b3}	$4d_{xy}$	$4d_{xz}$	$4d_{yz}$	$4d_{x^2-y^2}$	$4d_{z^2}$	
$^2T_1$	2.00	0.00	2	2	2	2	2	u	0	0	2	0	2	0	0	0	0	0	0	0	0	0	61
			2	2	2	2	2	u	0	0	0	0	2	0	0	2	0	0	0	0	0	0	22
^2E	2.03	0.00	2	2	2	2	2	u	0	0	d	0	2	0	u	0	0	0	0	0	0	0	84
$^2T_1$	2.04	0.00	2	2	2	2	2	2	2	0	0	0	0	2	0	u	0	0	0	0	0	0	34
			2	2	2	2	2	0	0	0	2	0	2	0	0	u	0	0	0	0	0	0	51
$^2T_1$	2.08	0.00	2	2	2	2	2	2	0	0	u	0	2	0	0	0	0	0	0	0	0	0	55
			2	2	2	2	2	0	0	0	u	0	2	0	0	2	0	0	0	0	0	0	30
^2E	2.11	0.00	2	2	2	2	2	u	0	0	u	0	2	0	0	d	d	0	0	0	0	0	84
$^2T_2$	2.86	0.00	2	2	2	2	2	2	2	0	0	0	0	2	0	u	0	0	0	0	0	0	49
			2	2	2	2	2	0	0	0	2	0	2	0	0	u	0	0	0	0	0	0	29
$^2T_2$	2.99	0.00	2	2	2	2	2	2	0	0	u	0	2	0	0	0	0	0	0	0	0	0	27
			2	2	2	2	2	0	0	0	u	0	2	0	0	2	0	0	0	0	0	0	49
$^2T_2$	3.03	0.00	2	2	2	2	61	u	0	0	2	0	2	0	0	0	0	0	0	0	0	0	61
			2	2	2	2	2	u	0	0	0	0	2	0	0	2	0	0	0	0	0	0	22

Table S6. Weights of the five most important spin-orbit-free states for each spin-orbit state. Q_1–Q_8 refer to the quartet excited states in Table S4, ordered by increasing energy. D_1–D_8 refer to the doublet excited states in Table S5, ordered by increasing energy. All results were obtained at RASPT2 level of theory in Molcas 8.4.

SO	SF	Weight	SF	Weight	SF	Weight	SF	Weight	SF	Weight
1	GS	0.9996	Q_2	0.0001	D_6	0.0001	Q_1	0.0001	D_8	0.0000
2	GS	0.9996	Q_2	0.0001	D_6	0.0001	Q_1	0.0001	D_8	0.0000
3	GS	0.9996	Q_3	0.0001	Q_1	0.0001	D_7	0.0001	D_8	0.0000
4	GS	0.9996	Q_3	0.0001	Q_1	0.0001	D_7	0.0001	D_8	0.0000
5	D_1	0.9231	D_3	0.0431	D_2	0.0278	D_4	0.003	Q_2	0.0013
6	D_1	0.9231	D_3	0.0431	D_2	0.0278	D_4	0.003	Q_2	0.0013
7	D_2	0.8203	D_3	0.1488	D_4	0.025	D_1	0.0042	Q_1	0.0010
8	D_2	0.8203	D_3	0.1488	D_4	0.025	D_1	0.0042	Q_1	0.0010
9	D_3	0.8051	D_2	0.1260	D_1	0.0637	D_4	0.0022	Q_1	0.0008
10	D_3	0.8051	D_2	0.1260	D_1	0.0637	D_4	0.0022	Q_1	0.0008
11	D_4	0.9574	D_2	0.0230	D_5	0.0106	D_1	0.0055	Q_3	0.0010
12	D_4	0.9574	D_2	0.0230	D_5	0.0106	D_1	0.0055	Q_3	0.0010
13	D_5	0.9857	D_4	0.0101	Q_2	0.0009	D_3	0.0007	D_6	0.0007
14	D_5	0.9857	D_4	0.0101	Q_2	0.0009	D_3	0.0007	D_6	0.0007
15	Q_1	0.7762	Q_2	0.1996	Q_3	0.0178	D_6	0.0049	D_2	0.0007
16	Q_1	0.7762	Q_2	0.1996	Q_3	0.0178	D_6	0.0049	D_2	0.0007
17	Q_1	0.9212	Q_2	0.0423	Q_3	0.0297	D_6	0.0029	D_7	0.0029

18	Q_1	0.9212	Q_2	0.0423	Q_3	0.0297	D_6	0.0029	D_7	0.0029
19	Q_2	0.9599	Q_1	0.0330	Q_3	0.0029	D_1	0.0014	D_6	0.0011
20	Q_2	0.9599	Q_1	0.0330	Q_3	0.0029	D_1	0.0014	D_6	0.0011
21	Q_2	0.7856	Q_1	0.2053	Q_3	0.0049	D_6	0.0017	D_5	0.0010
22	Q_2	0.7856	Q_1	0.2053	Q_3	0.0049	D_6	0.0017	D_5	0.0010
23	Q_3	0.9879	Q_1	0.0063	D_8	0.0014	Q_2	0.0009	D_1	0.0009
24	Q_3	0.9879	Q_1	0.0063	D_8	0.0014	Q_2	0.0009	D_1	0.0009
25	Q_3	0.9473	Q_1	0.0483	12	0.0021	D_4	0.0009	D_2	0.0006
26	Q_3	0.9473	Q_1	0.0483	12	0.0021	D_4	0.0009	D_2	0.0006
27	D_6	0.9846	Q_2	0.0073	Q_1	0.0038	D_7	0.0014	Q_4	0.0009
28	D_6	0.9846	Q_2	0.0073	Q_1	0.0038	D_7	0.0014	Q_4	0.0009
29	D_7	0.9083	D_8	0.0814	Q_3	0.0049	Q_1	0.0018	D_6	0.0014
30	D_7	0.9083	D_8	0.0814	Q_3	0.0049	Q_1	0.0018	D_6	0.0014
31	D_8	0.9124	D_7	0.0819	Q_5	0.0022	Q_4	0.0013	Q_3	0.0010
32	D_8	0.9124	D_7	0.0819	Q_5	0.0022	Q_4	0.0013	Q_3	0.0010
33	Q_4	0.9501	Q_5	0.0471	D_8	0.0016	Q_3	0.0007	Q_1	0.0003
34	Q_4	0.9501	Q_5	0.0471	D_8	0.0016	Q_3	0.0007	Q_1	0.0003
35	Q_4	0.9916	Q_5	0.0060	D_6	0.0011	Q_1	0.0005	D_8	0.0004
36	Q_4	0.9916	Q_5	0.0060	D_6	0.0011	Q_1	0.0005	D_8	0.0004
37	Q_5	0.9918	Q_4	0.0063	Q_6	0.0006	D_8	0.0004	D_7	0.0004
38	Q_5	0.9918	Q_4	0.0063	Q_6	0.0006	D_8	0.0004	D_7	0.0004
39	Q_5	0.9504	Q_4	0.0474	D_8	0.0010	Q_2	0.0005	Q_6	0.0002

40	Q_5	0.9504	Q_4	0.0474	D_8	0.0010	Q_2	0.0005	Q_6	0.0002
41	Q_6	0.9987	Q_2	0.0003	Q_5	0.0003	D_6	0.0002	Q_3	0.0002
42	Q_6	0.9987	Q_2	0.0003	Q_5	0.0003	D_6	0.0002	Q_3	0.0002
43	Q_6	0.9980	D_7	0.0009	Q_5	0.0005	Q_3	0.0003	D_4	0.0001
44	Q_6	0.9980	D_7	0.0009	Q_5	0.0005	Q_3	0.0003	D_4	0.0001
45	Q_7	1.0000	D_7	0.0000	Q_3	0.0000	Q_6	0.0000	Q_5	0.0000
46	Q_7	1.0000	Q_3	0.0000	D_6	0.0000	Q_4	0.0000	Q_5	0.0000
47	Q_7	1.0000	Q_3	0.0000	D_6	0.0000	Q_4	0.0000	Q_5	0.0000
48	Q_7	1.0000	D_7	0.0000	Q_3	0.0000	Q_6	0.0000	Q_5	0.0000

References

1. M. J. Frisch, G. W. Trucks, H. B. Schlegel, G. E. Scuseria, M. A. Robb, J. R. Cheeseman, G. Scalmani, V. Barone, G. A. Petersson, H. Nakatsuji, X. Li, M. Caricato, A. V. Marenich, J. Bloino, B. G. Janesko, R. Gomperts, B. Mennucci, H. P. Hratchian, J. V. Ortiz, A. F. Izmaylov, J. L. Sonnenberg, Williams, F. Ding, F. Lipparini, F. Egidi, J. Goings, B. Peng, A. Petrone, T. Henderson, D. Ranasinghe, V. G. Zakrzewski, J. Gao, N. Rega, G. Zheng, W. Liang, M. Hada, M. Ehara, K. Toyota, R. Fukuda, J. Hasegawa, M. Ishida, T. Nakajima, Y. Honda, O. Kitao, H. Nakai, T. Vreven, K. Throssell, J. A. Montgomery Jr., J. E. Peralta, F. Ogliaro, M. J. Bearpark, J. J. Heyd, E. N. Brothers, K. N. Kudin, V. N. Staroverov, T. A. Keith, R. Kobayashi, J. Normand, K. Raghavachari, A. P. Rendell, J. C. Burant, S. S. Iyengar, J. Tomasi, M. Cossi, J. M. Millam, M. Klene, C. Adamo, R. Cammi, J. W. Ochterski, R. L. Martin, K. Morokuma, O. Farkas, J. B. Foresman and D. J. Fox, Gaussian 16 Rev. C.02, 2016.
2. A. D. Becke, Density-functional thermochemistry. III. The role of exact exchange, *J. Chem. Phys.*, 1993, **98**, 5648-5652.
3. A. D. Becke, Density-functional exchange-energy approximation with correct asymptotic behavior, *Phys. Rev. A.*, 1988, **38**, 3098.
4. J. Guthmuller and L. González, Simulation of the resonance Raman intensities of a ruthenium–palladium photocatalyst by time dependent density functional theory, *Phys. Chem. Chem. Phys.*, 2010, **12**, 14812-14821.
5. C. Lee, W. Yang and R. G. Parr, Development of the Colle-Salvetti correlation-energy formula into a functional of the electron density, *Phys. Rev. B.*, 1988, **37**, 785.
6. G. E. Shillito, T. B. Hall, D. Preston, P. Traber, L. Wu, K. E. Reynolds, R. Horvath, X. Z. Sun, N. T. Lucas and J. D. Crowley, Dramatic alteration of 3ILCT lifetimes using ancillary ligands in [Re (L)(CO) 3 (phen-TPA)] n+ complexes: an integrated spectroscopic and theoretical study, *J. Am. Chem. Soc.*, 2018, **140**, 4534-4542.
7. G. E. Shillito, D. Preston, P. Traber, J. Steinmetzer, C. J. McAdam, J. D. Crowley, P. Wagner, S. Kupfer and K. C. Gordon, Excited-state switching frustrates the tuning of properties in triphenylamine-donor-ligand rhenium (I) and platinum (II) complexes, *Inorg. Chem.*, 2020, **59**, 6736-6746.
8. F. Weigend, Accurate Coulomb-fitting basis sets for H to Rn, *Phys. Chem. Chem. Phys.*, 2006, **8**, 1057-1065.
9. F. Weigend and R. Ahlrichs, Balanced basis sets of split valence, triple zeta valence and quadruple zeta valence quality for H to Rn: Design and assessment of accuracy, *Phys. Chem. Chem. Phys.*, 2005, **7**, 3297-3305.
10. A. V. Marenich, C. J. Cramer and D. G. Truhlar, Universal solvation model based on solute electron density and on a continuum model of the solvent defined by the bulk dielectric constant and atomic surface tensions, *J. Phys. Chem. B.*, 2009, **113**, 6378-6396.
11. B. Mennucci, C. Cappelli, C. A. Guido, R. Cammi and J. Tomasi, Structures and properties of electronically excited chromophores in solution from the polarizable continuum model coupled to the time-dependent density functional theory, *J. Phys. Chem. A.*, 2009, **113**, 3009-3020.
12. S. Grimme, S. Ehrlich and L. Goerigk, Effect of the damping function in dispersion corrected density functional theory, *J. Comput. Chem.*, 2011, **32**, 1456-1465.
13. D. Feller, The role of databases in support of computational chemistry calculations, *J. Comput. Chem.*, 1996, **17**, 1571-1586.
14. K. L. Schuchardt, B. T. Didier, T. Elsethagen, L. Sun, V. Gurumoorthi, J. Chase, J. Li and T. L. Windus, Basis set exchange: a community database for computational sciences, *J. Chem. Inf. Model.*, 2007, **47**, 1045-1052.
15. A. K. Wilson, D. E. Woon, K. A. Peterson and T. H. Dunning Jr, Gaussian basis sets for use in correlated molecular calculations. IX. The atoms gallium through krypton, *J. Chem. Phys.*, 1999, **110**, 7667-7676.
16. P. Å. Malmqvist, K. Pierloot, A. R. M. Shahi, C. J. Cramer and L. Gagliardi, The restricted active space followed by second-order perturbation theory method: Theory and application to the study of CuO2 and Cu2O2 systems, *J. Chem. Phys.*, 2008, **128**.

17. P. Å. Malmqvist, A. Rendell and B. O. Roos, The restricted active space self-consistent-field method, implemented with a split graph unitary group approach, *J. Phys. Chem.*, 1990, **94**, 5477-5482.
18. J. Olsen, B. O. Roos, P. Jo/rgensen and H. J. r. A. Jensen, Determinant based configuration interaction algorithms for complete and restricted configuration interaction spaces, *J. Chem. Phys.*, 1988, **89**, 2185-2192.
19. K. D. Vogiatzis, G. Li Manni, S. J. Stoneburner, D. Ma and L. Gagliardi, Systematic expansion of active spaces beyond the CASSCF limit: A GASSCF/SplitGAS benchmark study, *J. Chem. Theory Comput.*, 2015, **11**, 3010-3021.
20. F. Aquilante, J. Autschbach, R. K. Carlson, L. F. Chibotaru, M. G. Delcey, L. De Vico, I. Fdez. Galván, N. Ferré, L. M. Frutos, L. Gagliardi, M. Garavelli, A. Giussani, C. E. Hoyer, G. Li Manni, H. Lischka, D. Ma, P. Å. Malmqvist, T. Müller, A. Nenov, M. Olivucci, T. B. Pedersen, D. Peng, F. Plasser, B. Pritchard, M. Reiher, I. Rivalta, I. Schapiro, J. Segarra-Martí, M. Stenrup, D. G. Truhlar, L. Ungur, A. Valentini, S. Vancoillie, V. Veryazov, V. P. Vysotskiy, O. Weingart, F. Zapata and R. Lindh, Molcas 8: New capabilities for multiconfigurational quantum chemical calculations across the periodic table, *J. Comput. Chem.*, 2016, **37**, 506-541.
21. T. Nakajima and K. Hirao, The Douglas–Kroll–Hess Approach, *Chem. Rev.*, 2012, **112**, 385-402.
22. A. Wolf, M. Reiher and B. A. Hess, The generalized douglas–kroll transformation, *J. Chem. Phys.*, 2002, **117**, 9215-9226.
23. K. Shizu and H. Kaji, Theoretical determination of rate constants from excited states: Application to benzophenone, *J. Phys. Chem. A.*, 2021, **125**, 9000-9010.
24. D. P. Craig and T. Thirunamachandran, *Molecular quantum electrodynamics: an introduction to radiation-molecule interactions*, Courier Corporation, 1998.
25. L. Martínez, R. Andrade, E. G. Birgin and J. M. Martínez, PACKMOL: A package for building initial configurations for molecular dynamics simulations, *J. Comput. Chem.*, 2009, **30**, 2157-2164.
26. J. Wang, R. M. Wolf, J. W. Caldwell, P. A. Kollman and D. A. Case, Development and testing of a general amber force field, *J. Comput. Chem.*, 2004, **25**, 1157-1174.
27. X. He, V. H. Man, W. Yang, T.-S. Lee and J. Wang, A fast and high-quality charge model for the next generation general AMBER force field, *J. Chem. Phys.*, 2020, **153**.
28. J. Wang, W. Wang, P. A. Kollman and D. A. Case, Automatic atom type and bond type perception in molecular mechanical calculations, *J. Mol. Graph. Model.*, 2006, **25**, 247-260.
29. W. D. Cornell, P. Cieplak, C. I. Bayly, I. R. Gould, K. M. Merz, D. M. Ferguson, D. C. Spellmeyer, T. Fox, J. W. Caldwell and P. A. Kollman, A second generation force field for the simulation of proteins, nucleic acids, and organic molecules, *J. Am. Chem. Soc.*, 1995, **117**, 5179-5197.
30. D. A. Case, H. M. Aktulga, K. Belfon, I. Ben-Shalom, S. R. Brozell, D. S. Cerutti, T. E. Cheatham III, V. W. D. Cruzeiro, T. A. Darden and R. E. Duke, *Amber 2021*, University of California, San Francisco, 2021.
31. M. Kowalewski and P. Seeber, Sustainable packaging of quantum chemistry software with the Nix package manager, *Int. J. Quantum Chem.*, 2022, **122**, e26872.
32. J. Hutter, M. Iannuzzi, F. Schiffmann and J. VandeVondele, cp2k: atomistic simulations of condensed matter systems, *Wiley Interdiscip. Rev. Comput. Mol. Sci.*, 2014, **4**, 15-25.
33. J. VandeVondele, M. Krack, F. Mohamed, M. Parrinello, T. Chassaing and J. Hutter, Quickstep: Fast and accurate density functional calculations using a mixed Gaussian and plane waves approach, *Comput. Phys. Commun.*, 2005, **167**, 103-128.
34. B. Miehlich, A. Savin, H. Stoll and H. Preuss, Results obtained with the correlation energy density functionals of becke and Lee, Yang and Parr, *Chem. Phys. Lett.*, 1989, **157**, 200-206.
35. J. VandeVondele and J. Hutter, Gaussian basis sets for accurate calculations on molecular systems in gas and condensed phases, *J. Chem. Phys.*, 2007, **127**.
36. N. E. Schultz, Y. Zhao and D. G. Truhlar, Density functionals for inorganometallic and organometallic chemistry, *J. Phys. Chem. A.*, 2005, **109**, 11127-11143.

37. S. M. Tekarli, M. L. Drummond, T. G. Williams, T. R. Cundari and A. K. Wilson, Performance of density functional theory for 3d transition metal-containing complexes: utilization of the correlation consistent basis sets, *J. Phys. Chem. A.*, 2009, **113**, 8607-8614.

38. S. Goedecker, M. Teter and J. Hutter, Separable dual-space Gaussian pseudopotentials, *Phys. Rev. B.*, 1996, **54**, 1703.

39. C. Hartwigsen, S. Gœdecker and J. Hutter, Relativistic separable dual-space Gaussian pseudopotentials from H to Rn, *Phys. Rev. B.*, 1998, **58**, 3641.

40. M. Krack, Pseudopotentials for H to Kr optimized for gradient-corrected exchange-correlation functionals, *Theor. Chem. Acc.*, 2005, **114**, 145-152.

41. G. Bussi, D. Donadio and M. Parrinello, Canonical sampling through velocity rescaling, *J. Chem. Phys.*, 2007, **126**.

42. L. Hu, M. Farrokhnia, J. Heimdal, S. Shleev, L. Rulisek and U. Ryde, Reorganization energy for internal electron transfer in multicopper oxidases, *J. Phys. Chem. B.*, 2011, **115**, 13111-13126.

43. J. Blumberger, Free energies for biological electron transfer from QM/MM calculation: method, application and critical assessment, *Phys. Chem. Chem. Phys.*, 2008, **10**, 5651-5667.

44. A. Warshel, Dynamics of reactions in polar solvents. Semiclassical trajectory studies of electron-transfer and proton-transfer reactions, *J. Phys. Chem.*, 1982, **86**, 2218-2224.

45. J. Blumberger and M. Sprik, Quantum versus classical electron transfer energy as reaction coordinate for the aqueous Ru2+/Ru3+ redox reaction, *Theor. Chem. Acc.*, 2006, **115**, 113-126.

46. M. Tachiya, Relation between the electron-transfer rate and the free energy change of reaction, *J. Phys. Chem.*, 1989, **93**, 7050-7052.

47. Y. Tateyama, J. Blumberger, M. Sprik and I. Tavernelli, Density-functional molecular-dynamics study of the redox reactions of two anionic, aqueous transition-metal complexes, *J. Chem. Phys.*, 2005, **122**.

48. R. A. Marcus, On the theory of oxidation-reduction reactions involving electron transfer. I, *J. Chem. Phys.*, 1956, **24**, 966-978.

49. R. A. Marcus, On the theory of electron-transfer reactions. VI. Unified treatment for homogeneous and electrode reactions, *J. Chem. Phys.*, 1965, **43**, 679-701.

50. R. A. Marcus, Electron transfer reactions in chemistry: theory and experiment (Nobel lecture), *Angewandte Chemie International Edition in English*, 1993, **32**, 1111-1121.

51. A. Koch, D. Kinzel, F. Dröge, S. Gräfe and S. Kupfer, Photochemistry and Electron Transfer Kinetics in a Photocatalyst Model Assessed by Marcus Theory and Quantum Dynamics, *J. Phys. Chem. C*, 2017, **121**, 16066-16078.

52. G. Yang, G. E. Shillito, C. Zens, B. Dietzek-Ivanšić and S. Kupfer, The three kingdoms—Photoinduced electron transfer cascades controlled by electronic couplings, *J. Chem. Phys.*, 2023, **159**.

53. G. Yang, L. Blechschmidt, L. Zedler, C. Zens, K. Witas, M. Schmidt, B. Esser, S. Rau, G. E. Shillito, B. Dietzek-Ivanšić and S. Kupfer, Excited State Branching Processes in a Ru(II)-Based Donor–Acceptor–Donor System, *Chemistry – A European Journal*, 2025, **31**, e202404671.

54. M. Staniszewska, S. Kupfer and J. Guthmuller, Theoretical investigation of the electron-transfer dynamics and photodegradation pathways in a hydrogen-evolving ruthenium–palladium photocatalyst, *Eur. J. Chem.*, 2018, **24**, 11166-11176.

55. G. E. Shillito, S. Rau and S. Kupfer, Plugging the 3MC sink in RuII-Based photocatalysts, *ChemCatChem*, 2023, **15**, e202201489.

56. M. Staniszewska, S. Kupfer and J. Guthmuller, Effect of the catalytic center on the electron transfer dynamics in hydrogen-evolving ruthenium-based photocatalysts investigated by theoretical calculations, *J. Phys. Chem. C.*, 2019, **123**, 16003-16013.

57. C. Zens, C. Friebe, U. S. Schubert, M. Richter and S. Kupfer, Tailored charge transfer kinetics in precursors for organic radical batteries: A joint synthetic-theoretical approach, *ChemSusChem.*, 2023, **16**, e202201679.

58. C. Scarborough, S. Sproules, T. Weyermuller, S. DeBeer and K. Wieghardt, *Electronic and Molecular Structures of the Members of the Electron Transfer Series*, SLAC National Accelerator Lab., Menlo Park, CA (United States), 2013.
